# Stochastic cloud fluctuations drive Arctic winter radiative bistability

**DOI:** 10.1126/sciadv.aeh3823

**Published:** 2026-09-30

**Authors:** Jung-Sub Lim, Graham Feingold

**Affiliations:** ^1^ NOAA Chemical Sciences Laboratory, Boulder, CO, USA.; ^2^ Cooperative Institute for Research in Environmental Sciences, University of Colorado, Boulder, CO, USA.

## Abstract

In Arctic winter, the boundary layer occupies two preferred radiative states, a cold, clear state and a heat-trapping, cloudy state, that strongly regulate surface energy loss over sea ice. Yet, reanalyses and climate models are often biased toward a single intermediate state. Using a stochastic differential equation framework applied to 27 years of high-resolution observations, we show that the observed bistability is not sustained by multiple deterministic equilibria: The mean restoring tendency is effectively single-well, while background meteorology alone cannot explain rapid transitions. Instead, the two regimes are sustained by sharply localized, state-dependent noise in the transition range. This noise structure emerges when subhourly fluctuations in liquid water path are radiatively amplified by the nonlinear saturation of longwave emissivity. Our results provide a dynamical explanation for this model bias and identify unresolved fast cloud variability as a key source of uncertainty in Arctic winter climate projections.

## INTRODUCTION

Arctic near-surface temperatures are rising at nearly four times the global mean rate (*1*, *2*). This amplification peaks in winter (*3*, *4*), when the absence of sunlight leaves the surface energy budget dominated by longwave exchange. Under these conditions, low-level clouds become a primary regulator of surface cooling by re-emitting thermal radiation downward, reducing net longwave heat loss and shaping winter surface temperature variability and thermodynamic sea-ice growth (*5*).

Wintertime observations further show that Arctic mixed-phase clouds, which contain both supercooled liquid water and ice, organize the boundary layer into two preferred radiative regimes (*6*–*8*): a radiatively clear regime and an opaque cloudy regime. In surface net longwave flux ($F_{\text{LW},\text{net}}$), this two-regime structure appears as rapid transitions on subhourly to multihour timescales together with residence times lasting hours to days (*6*, *8*). Regime occupancy therefore exerts a first-order control on the winter surface energy balance over sea ice (*9*).

What maintains this radiative bistability remains unclear. Mixed-phase microphysics would be expected to favor rapid glaciation through the Wegener-Bergeron-Findeisen mechanism on timescales of about an hour, a process that would rapidly deplete liquid water and transition the system to a clear state, yet low-level mixed-phase clouds can persist for days (*10*). Synoptic and large-scale dynamical regimes modulate the background environment (*6*, *7*, *11*), but these comparatively slow variations alone do not explain the sharp separation between radiative states. The central question is therefore whether the observed regimes reflect distinct deterministic equilibria or are maintained by fast cloud-scale variability.

This question has become more important because large-scale models and reanalyses often fail to reproduce the observed two-regime structure. Despite Arctic winter bimodality being documented across multiple sites (*12*–*14*), reanalysis products such as the ECMWF Reanalysis version 5 (ERA5) (*15*) often collapse toward a single intermediate radiative state (*8*, *16*). At the same time, observations indicate recent shifts toward more radiatively opaque winter clouds (*17*). Together, these results point to a missing dynamical ingredient that cannot be diagnosed from mean-state behavior alone.

A stochastic framework provides a reduced description that separates the average evolution of a state variable from fast variability around that average. In this framework, the average tendency is represented by the drift, while the amplitude of unresolved fast variability, or noise, is represented by the state-dependent diffusion coefficient. When this noise amplitude depends on the state, distinct regimes can arise even when the drift contains only a single attracting equilibrium (*18*–*20*). Fast cloud-scale variability can therefore act as an active dynamical ingredient, rather than merely as residual noise.

Here, we provide a dynamical framework for the noise-induced origin of this Arctic winter radiative bistability, in which the clear and cloudy regimes are maintained by rapid cloud-driven fluctuations. We model $F_{\text{LW},\text{net}}$ as a one-dimensional stochastic process and infer its drift and diffusion using nonparametric Kramers-Moyal estimation (*21*, *22*) from a 27-year high-resolution record at Utqiaġvik. We then test the robustness of the inferred structure using independent records from Ny-Ålesund and the Multidisciplinary drifting Observatory for the Study of Arctic Climate (MOSAiC) campaign (*23*). Across these independent Arctic records, we find the same qualitative dynamical structure: The observed bistability is not sustained by multiple deterministic equilibria. Instead, the inferred drift has a single equilibrium, while a localized enhancement of cloud-driven, state-dependent diffusion in the transition range separates the clear and cloudy regimes. This reduced description suggests that the unimodal bias in reanalyses and climate models arises from their underrepresentation of the unresolved subhourly cloud variability associated with maintaining the two winter radiative regimes.

## RESULTS

### The Arctic radiative bimodality

Ground-based observations from Utqiaġvik show that wintertime $F_{\text{LW},\text{net}}$ concentrates near two distinct values (Fig. 1C): A radiatively clear regime with strong surface cooling ($F_{\text{LW},\text{net}}\approx -45Wm^{-2}$) and an opaque cloudy regime with strongly reduced longwave heat loss ($F_{\text{LW},\text{net}}\approx -5Wm^{-2}$). The corresponding time series exhibits extended residence near each mode, with rapid transitions between them (Fig. 1A).

**Fig. 1.**
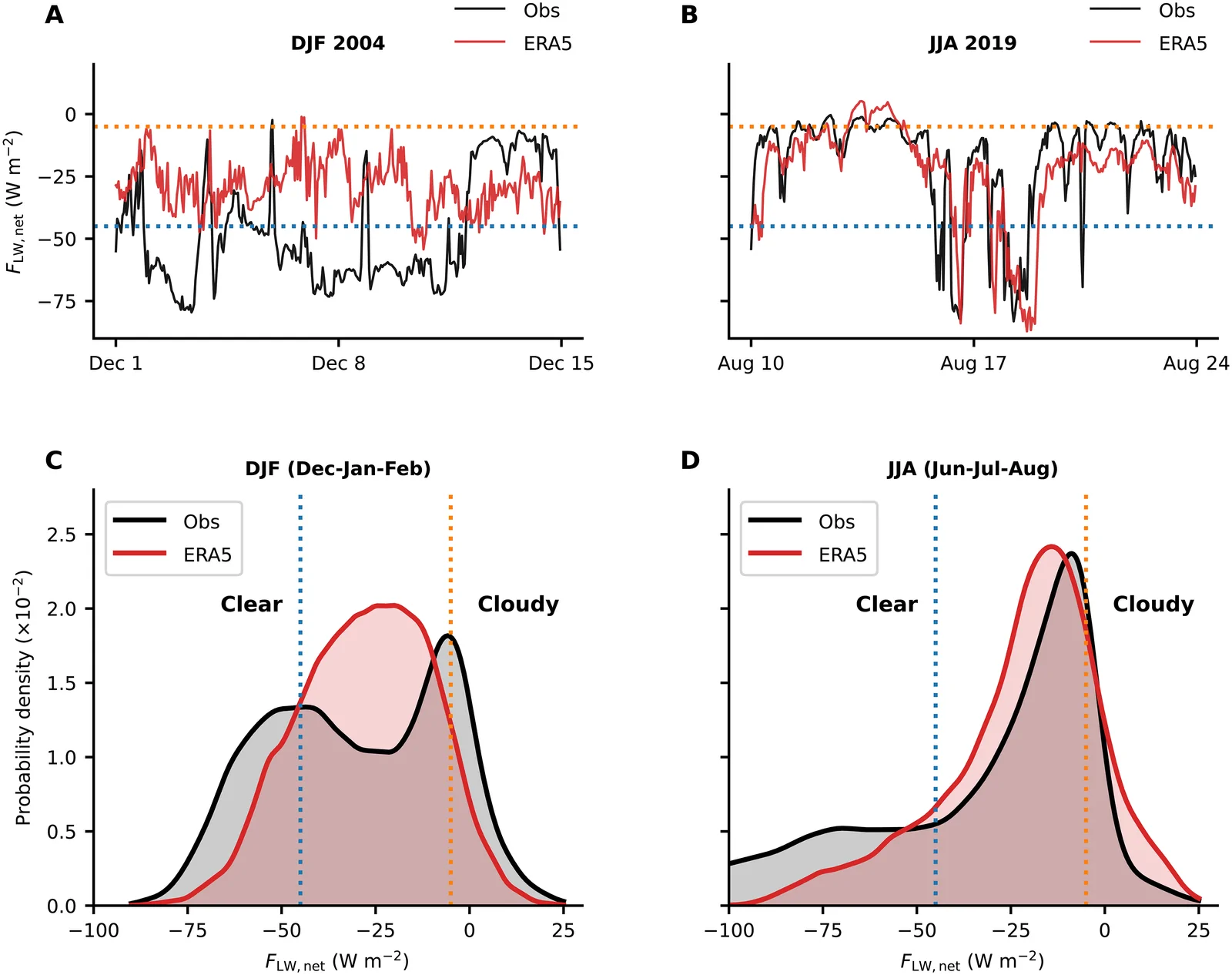
Bimodality of net surface longwave radiation $F_{\text{LW},\text{net}}$ in observations versus ERA5. (**A** and **B**) Representative time series of $F_{\text{LW},\text{net}}$ for winter (A) and summer (B) from ground-based observations (black) and ERA5 (red). (**C** and **D**) Corresponding probability density functions (PDFs) for winter (DJF) and summer (JJA). Dotted lines indicate the characteristic clear (blue) and cloudy (orange) regime locations used as references in subsequent analyses. Distributions use the full multiyear record (27 winters, 1998–2025) and the concurrent ERA5 period.

Conversely, ERA5 fails to reproduce this bimodal structure. Instead, the winter [December, January, and February (DJF)] probability density function (PDF) collapses toward a single intermediate peak ($F_{\text{LW},\text{net}}\approx -28Wm^{-2}$), and the time series fluctuates persistently without locking into the observed regimes (Fig. 1A) (*24*, *25*). In summer [June, July, and August (JJA)], however, both observations and ERA5 are predominantly unimodal with a similar peak near $F_{\text{LW},\text{net}}\approx -10Wm^{-2}$ (Fig. 1D) and correlated time series (Fig. 1B), indicating that this unimodal bias is specific to polar night.

### Noise-induced bistability with a single-equilibrium drift

To identify what drives these winter regimes and ERA5’s unimodal bias, we model the net surface longwave flux, $x_{t}\equiv F_{\text{LW},\text{net}}\left(t\right)$, using an effective one-dimensional Itô stochastic differential equation (SDE) with state-dependent drift and diffusion, written in Langevin form

$$dx_{t}=a\left(x_{t}\right)dt+\sqrt{2D\left(x_{t}\right)}dW_{t}$$ (1)

where a(x) is the drift coefficient, representing the average tendency of $F_{\text{LW},\text{net}}$, and D(x) is the diffusion coefficient, representing the state-dependent strength of fast stochastic fluctuations around that tendency. The random forcing is represented by the Wiener process $W_{t}$ (*26*), while *D*(*x*) sets its amplitude through the factor $\sqrt{2D\left(x\right)}$. Equation 1 is therefore used as an effective leading-order drift-diffusion representation of the reduced process. The assumptions required for this reduction, and the possible influence of higher-order statistics, are assessed in the “Kramers-Moyal estimation of drift and diffusion” section.

We infer a(x) and D(x) from 27 DJF winters of $F_{\text{LW},\text{net}}$ observations at Utqiaġvik using Kramers-Moyal estimation (*21*). The inferred drift is well approximated by a linear restoring tendency toward a single intermediate equilibrium state in both observations and ERA5 (Fig. 2A)

$$a\left(x\right)\approx -\frac{1}{\tau_{\text{drift}}}\left(x-x_{\text{eq}}\right)$$ (2)

where $x_{\text{eq}}\approx -29Wm^{-2}$ [95% bootstrap confidence interval (CI) [−31 to −27]; bracketed ranges denote 95% CIs throughout] denotes the equilibrium state where $a\left(x_{\text{eq}}\right)=0$, and $\tau_{\text{drift}}\approx 6.7\text{hours}$ denotes the linear relaxation timescale. In this case, the drift alone would restore both clearer and cloudier states toward this intermediate radiative state, rather than maintaining two separate preferred regimes.

**Fig. 2.**
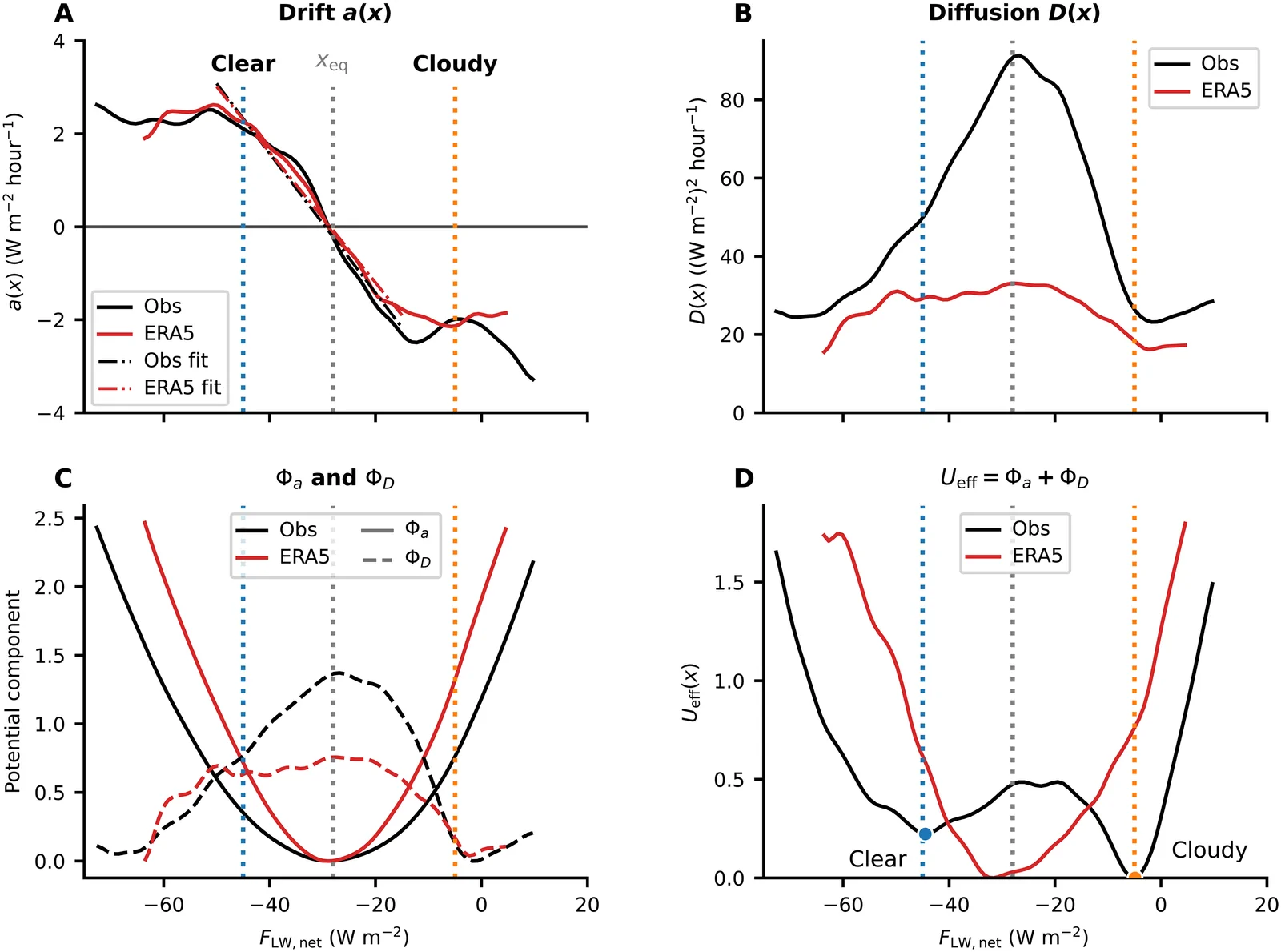
Drift, diffusion, and effective potential for net surface longwave radiation. (**A**) State-dependent drift *a*(*x*) inferred for the state variable $x\equiv F_{\text{LW},\text{net}}$ from observations (black) and ERA5 (red). Dashed lines show linear fits yielding relaxation timescales $\tau_{\text{drift}}\approx 6.7\text{hours}$ (95% CI [6.1 to 7.4]; observations, $R^{2}=0.96$) and $\tau_{\text{drift}}\approx 7.1\mathrm{hours}$ ([6.8 to 7.7]; ERA5, $R^{2}=0.99$). (**B**) State-dependent diffusion *D*(*x*). (**C**) Decomposition of the stationary effective potential into a drift contribution $\Phi_{a}$ and a diffusion contribution $\Phi_{D}$. Line color denotes the data source (observations: black, ERA5: red) and line style denotes the potential component ($\Phi_{a}$: solid, $\Phi_{D}$: dashed). (**D**) Resulting effective potential $U_{\text{eff}}\left(x\right)\equiv \Phi_{a}\left(x\right)+\Phi_{D}\left(x\right)$. Blue and orange circles mark the potential wells corresponding to the clear and cloudy regimes, respectively. Vertical dotted lines indicate the clear-regime location (blue), an intermediate reference state where $a\left(x_{\text{eq}}\right)=0$ (gray), and the cloudy-regime location (orange). All estimates derive from the full DJF observational record (27 winters, 1998–2025) and the concurrent ERA5 period.

This single-well drift does not preclude a bimodal stationary distribution. A bimodal distribution is often interpreted as evidence for two attracting states in the mean dynamics, or a deterministic double-well structure, but it can also arise when state-dependent fluctuations are sufficiently enhanced between the preferred regimes (*19*, *20*). In the Utqiaġvik observational record, this enhancement appears directly in the inferred diffusion coefficient: D(x) is strongly state-dependent, peaking in the transition range ($F_{\text{LW},\text{net}}\approx -40$ to $-10Wm^{-2}$) and weakening toward both regimes (Fig. 2B).

The effect of this peaked D(x) on the probability distribution can be estimated from the zero-current stationary solution of the one-dimensional Fokker-Planck equation associated with the DJF-conditioned effective dynamics in Eq. 1 (*26*), yielding the stationary density

$$p_{\text{st}}\left(x\right)\propto \frac{1}{D\left(x\right)}\text{exp}\left[\int_{x_{0}}^{x}\frac{a\left(y\right)}{D\left(y\right)}dy\right]$$ (3)

where $x_{0}$ is an arbitrary reference point. Here, $p_{\text{st}}\left(x\right)$ should be interpreted as an effective DJF-conditioned stationary density of the reduced stochastic process, rather than as a strictly time-invariant equilibrium distribution of the full multidecadal record. This quasi-stationary interpretation is supported by the moving-window, early/late-split, and detrending tests described in the “SDE model validation.”

Defining the effective potential, or probability landscape, as $U_{\text{eff}}\left(x\right)\equiv -\text{ln}p_{\text{st}}\left(x\right)$ yields the decomposition

$$U_{\text{eff}}\left(x\right)=\Phi_{a}\left(x\right)+\Phi_{D}\left(x\right)$$ (4)

with

$$\Phi_{a}\left(x\right)\equiv -\int_{x_{0}}^{x}\frac{a\left(y\right)}{D\left(y\right)}dy,\Phi_{D}\left(x\right)\equiv \text{ln}\left[D\left(x\right)\right]$$ (5)

In this effective potential, minima correspond to maxima of the stationary density and therefore identify the preferred clear and cloudy regimes, while maxima correspond to low-probability barriers between them. Because $\Phi_{D}\left(x\right)$ enters additively in Eq. 4, the transition-range peak in D(x) raises $U_{\text{eff}}\left(x\right)$ in that range, producing a barrier that separates the clear and cloudy regimes. Dynamically, these amplified stochastic fluctuations reduce residence time in the intermediate state, favoring rapid excursions toward either the clear or cloudy regime rather than sustained occupancy of the transition range. Consequently, $U_{\text{eff}}\left(x\right)$ exhibits two minima (Fig. 2D) corresponding to the clear and cloudy regimes, even though the drift has only a single equilibrium. The resulting stationary density $p_{\text{st}}\left(x\right)$ closely reproduces the observed bimodal PDF (Fig. 3B), and the inferred dynamics also reproduce the regime residence statistics (fig. S11B), confirming that the inferred SDE captures both the stationary distribution and the regime dynamics of wintertime $F_{\text{LW},\text{net}}$. Applying the same inference to independent records from Ny-Ålesund and MOSAiC yields a qualitatively similar drift-diffusion structure (fig. S1), indicating that the inferred structure is not specific to the Utqiaġvik record.

**Fig. 3.**
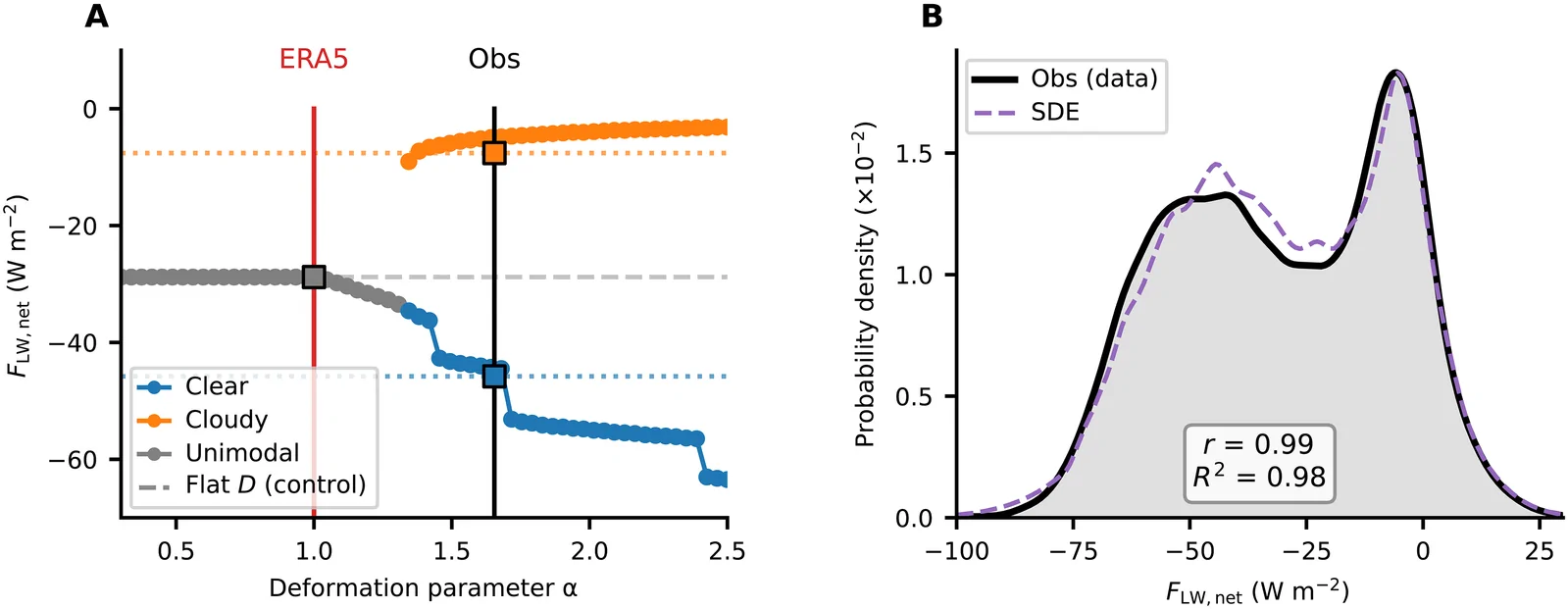
Noise-induced emergence of bimodality under controlled diffusion deformation. (**A**) Locations of stationary-PDF maxima as a function of deformation parameter α, which interpolates $D_{\alpha }\left(x\right)$ from the nearly flat ERA5 profile (α=1) to the observed state-dependent profile ($\alpha =\alpha_{\text{obs}}\approx 1.7$); beyond $\alpha_{\text{obs}}$ only the amplitude increases. The effective drift a(x) is held fixed throughout. Colored markers denote unimodal (gray), clear (blue), and cloudy (orange) regimes. The dashed gray curve shows a control in which spatially uniform *D* is amplified without introducing state dependence. (**B**) Comparison between the observed PDF from the 27-winter DJF record (black) and the stationary Fokker-Planck solution (purple dashed).

The unimodal ERA5 PDF arises primarily because ERA5 does not reproduce the transition-range enhancement of state-dependent diffusion seen in the observations. Although ERA5 captures a similar single-equilibrium drift (Fig. 2A), it strongly underestimates diffusion in the transition range, leaving D(x) nearly flat across radiative states (Fig. 2B). As a result, the diffusion term contributes little to the effective potential, so $U_{\text{eff}}\left(x\right)$ is controlled mainly by the drift contribution $\Phi_{a}\left(x\right)$. This produces a single-well effective potential (Fig. 2D) and, consequently, a unimodal PDF (Fig. 1C). Ideally, the observation-based and ERA5-based drift and diffusion estimates should be compared using the same sampling interval Δt. However, ERA5 does not provide data at a temporal resolution finer than 1 hour. To test whether this difference could arise from the different sampling intervals, we aggregate the 10-min observations to 1 hour for comparison with hourly ERA5 (fig. S2). This sensitivity test shows that the inferred drift is nearly unchanged by sampling interval (fig. S2A). Although the estimated diffusion magnitude decreases at coarser resolution, its peaked shape is retained (fig. S2B). Thus, temporal resolution alone cannot explain ERA5’s nearly state-independent diffusion.

We isolate the role of this state-dependent diffusion through a controlled SDE experiment. Because the inferred drift a(x) is nearly identical between observations and ERA5 (Fig. 2A), we hold a(x) fixed and continuously morph D(x) from the nearly flat ERA5 profile to the peaked observational profile (Fig. 3A). To separate the effect of D(x) shape from that of D(x) amplitude, we introduce a deformation parameter α. For $1\leq \alpha \leq \alpha_{\text{obs}}$, α linearly interpolates D(x) between ERA5 and the observed profile. For $\alpha >\alpha_{\text{obs}}$, we fix the observed shape and increase only the overall amplitude quadratically (“Kramers-Moyal estimation of drift and diffusion” section).

At α=1, the stationary density remains unimodal. As α increases, a noise-induced transition occurs: The intermediate maximum splits into distinct clear and cloudy regimes once α≳1.3 (Fig. 3A), recovering observed peak locations near $\alpha \approx \alpha_{\text{obs}}$. A control case in which the flat diffusion is uniformly amplified without introducing state dependence fails to produce this transition (Fig. 3A, gray dashed line). This indicates that ERA5’s unimodal bias stems from its near state-independent diffusion.

### Radiative sensitivity and LWP variability explain the peaked diffusion

We next ask what physical variability produces the transition-range diffusion peak that maintains the bimodal radiative distribution. In the reduced stochastic description, the drift describes the mean tendency of $F_{\text{LW},\text{net}}$, while the diffusion coefficient D(x) describes the amplitude of fast fluctuations around that mean tendency. On subhourly timescales, fluctuations in $F_{\text{LW},\text{net}}$ are dominated by cloud-driven variations in downwelling longwave radiation, whereas the upward longwave flux, $F_{\text{LW},\text{up}}$, evolves more slowly with surface temperature (fig. S3A). We therefore use the leading-order approximation

$$\mathrm{\delta x}\approx \delta F_{\text{LW},\text{down}}$$ (6)

Physically, these fast fluctuations in downwelling flux are driven by changes in cloud radiative properties. Since the longwave emissivity of Arctic clouds is determined primarily by the liquid water content, we link the variance D(x) directly to fluctuations in the vertical column-integrated liquid water content, liquid water path (LWP; Shupe-Turner multisensor retrieval; “Observational, reanalysis, and model datasets” section). This relationship is nonlinear: As LWP increases, cloud emissivity rises rapidly at first but eventually saturates at unity as clouds become thicker and behave like blackbodies. Consequently, the radiative sensitivity

$$S_{\text{LW}}\left(\text{LWP}\right)\equiv \frac{\partial F_{\text{LW},\text{net}}}{\partial \text{LWP}}$$ (7)

is not constant; it is high in thin clouds but decreases rapidly as clouds thicken (teal lines in Fig. 4A). In contrast, the conditional LWP variability $\sigma_{\text{LWP}}\left(x\right)\equiv \text{std}\left[\text{LWP}|x\right]$ tends to increase with increasing LWP (pink lines in Fig. 4A).

**Fig. 4.**
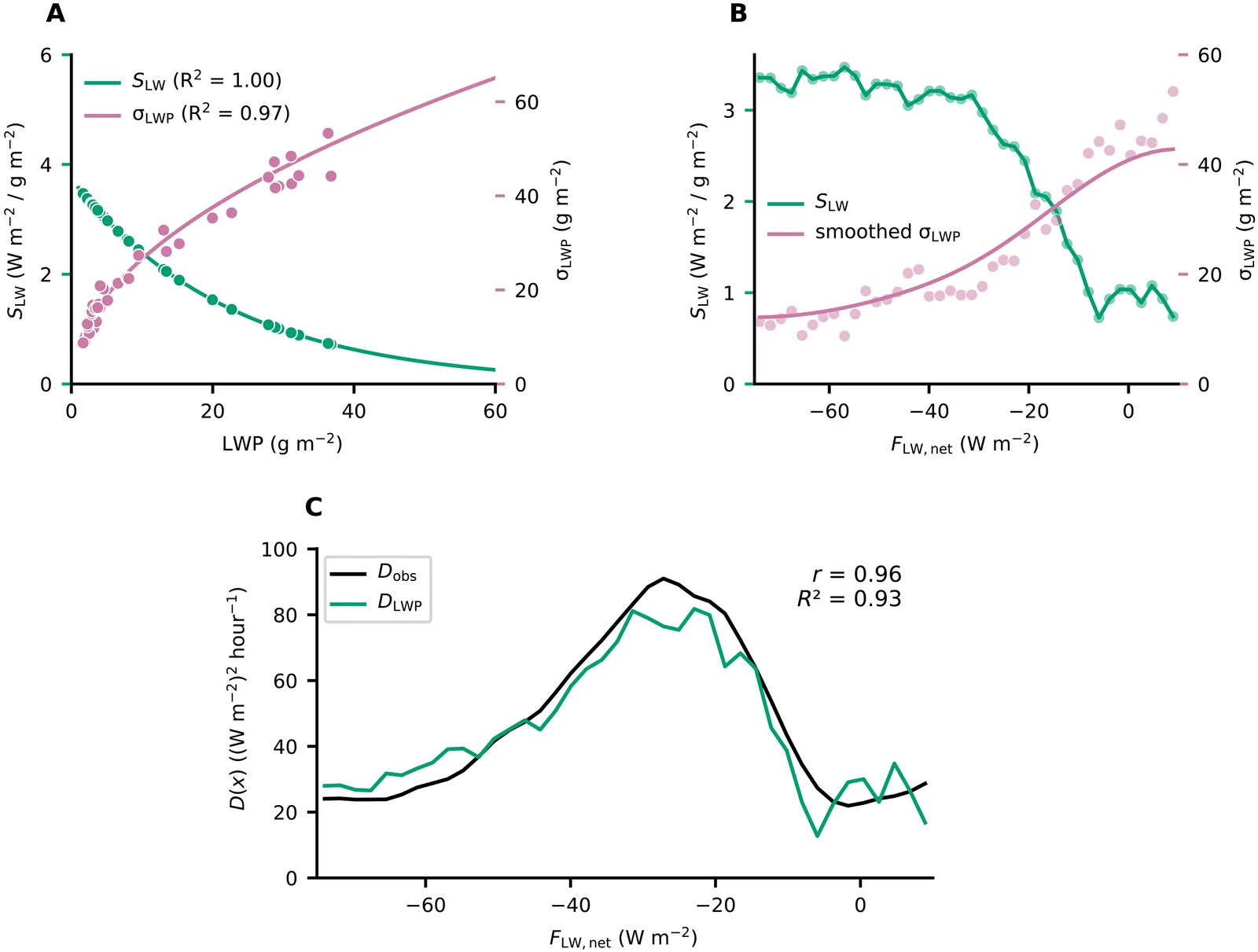
LWP-based microphysical closure for state-dependent diffusion. (**A**) Radiative sensitivity $S_{\text{LW}}\left(\text{LWP}\right)$ (teal; left axis) and LWP variability $\sigma_{\text{LWP}}$ (pink; right axis) as functions of LWP. (**B**) Radiative sensitivity and LWP variability expressed as functions of $F_{\text{LW},\text{net}}$, with $\sigma_{\text{LWP}}$ smoothed for clarity. (**C**) Comparison between the observed diffusion $D_{\text{obs}}$ (black) and the LWP-based estimate $D_{\text{LWP}}$ (teal), constructed as $D_{\text{LWP}}\left(x\right)=k_{\text{LW}}{\left[S_{\text{LW}}\left(x\right)\sigma_{\text{LWP}}\left(x\right)\right]}^{2}$. All quantities are estimated from the DJF 2004–2019 multivariate record.

Let $\text{δLWP}\left(t\right)\equiv \text{LWP}\left(t\right)-\bar{\text{LWP}}\left(x\right)$ denote the subhourly anomaly, $\text{var}\left[\text{δLWP}|x\right]=\sigma_{\text{LWP}}^{2}\left(x\right)$. Linearizing the radiative response around the conditional mean yields

$$\mathrm{\delta x}\left(t\right)\approx S_{\text{LW}}\left[\bar{\text{LWP}}\left(x\right)\right]\text{δLWP}\left(t\right)$$ (8)

Then, defining

$$S_{\text{LW}}\left(x\right)\equiv S_{\text{LW}}\left[\bar{\text{LWP}}\left(x\right)\right],g\left(x\right)\equiv S_{\text{LW}}\left(x\right)\sigma_{\text{LWP}}\left(x\right)$$ (9)

we find

$$\text{var}\left[\mathrm{\delta x}|x\right]\approx g^{2}\left(x\right)$$ (10)

Near-clear regimes possess weak $\sigma_{\text{LWP}}\left(x\right)$ (Fig. 4B), while thicker-cloud regimes (LWP ≳ 20 g $m^{-2}$; fig. S5) suffer suppressed $S_{\text{LW}}\left(x\right)$ due to emissivity saturation. This tradeoff forces D(x) to peak in the intermediate-flux transition range.

When these fluctuations are averaged over short windows, the effective diffusion scales with their conditional variance. Motivated by this, we estimate an LWP-based closure,

$$D_{\text{LWP}}\left(x\right)=k_{\text{LW}}{\left[S_{\text{LW}}\left(x\right)\sigma_{\text{LWP}}\left(x\right)\right]}^{2}$$ (11)

where $k_{\text{LW}}$ (hour^−1^) is the sole fitted prefactor, absorbing unresolved correlation-time effects. Despite its simplicity, $D_{\text{LWP}}\left(x\right)$ reproduces both the magnitude and the peaked state dependence of $D_{\text{obs}}\left(x\right)$ across the transition range (Fig. 4C), confirming that the diffusion peak arises where LWP fluctuations are most strongly amplified by cloud-radiative sensitivity.

### Seasonal decomposition of state-dependent diffusion

The winter analysis links radiative bistability to a localized, LWP-driven enhancement of state-dependent diffusion in the transition range. The disappearance of this bistability in summer therefore provides an independent test of the mechanism. Although ERA5’s wintertime $F_{\text{LW},\text{net}}$ distribution remains unimodal and misses the observed bimodality, its JJA distribution is much closer to observations (Fig. 1D). This contrast raises a mechanistic question: Does the closer summer agreement mean that ERA5’s cloud-liquid variability is better represented in JJA, or does the summer diffusion structure make the PDF less sensitive to errors in LWP-related variability?

We test this by inferring the JJA drift a(x) and diffusion D(x), and by diagnosing the physical controls on D(x). The inferred summer drift relaxes toward a more negative equilibrium flux in observations than in ERA5 (fig. S4A), while the diffusion difference is primarily one of magnitude rather than shape. Both observations and ERA5 exhibit larger diffusion toward the clear regime and smaller diffusion toward the cloudy regime (Fig. 5A). In contrast to winter, ERA5 mainly underestimates the magnitude of a broad summer diffusion structure, rather than missing a localized transition-range diffusion peak. Because this broad diffusion structure does not create a localized probability barrier in the effective potential, both observations and ERA5 retain a unimodal summer PDF despite differences in D(x). The summer PDF is therefore relatively insensitive to errors in the absolute magnitude of diffusion, allowing ERA5 to remain close to observations in distributional shape despite underestimating D(x) (Fig. 5A).

**Fig. 5.**
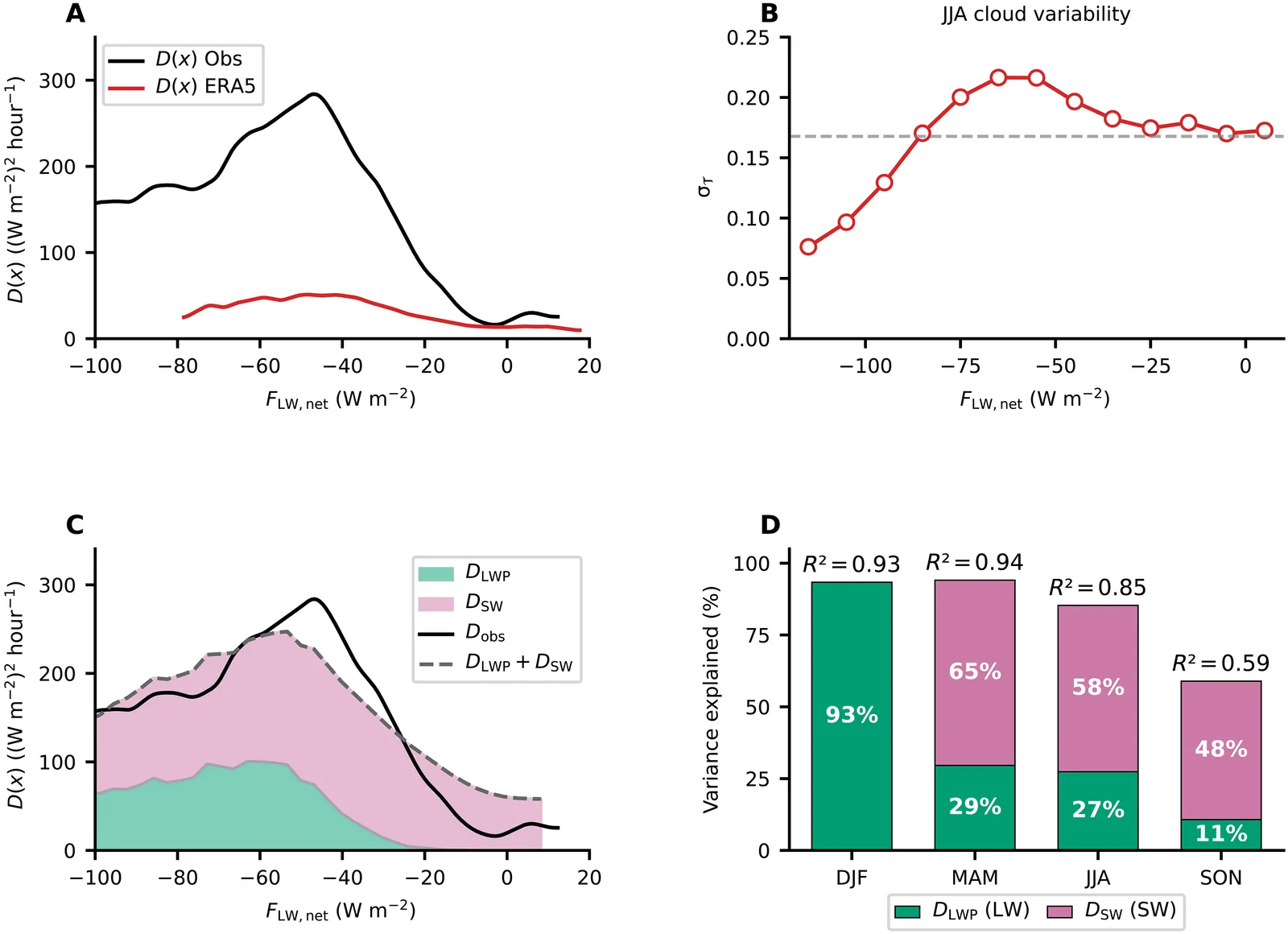
Seasonal decomposition of state-dependent diffusion into LWP- and shortwave-related contributions. (**A**) Summer (JJA) diffusion D(x) inferred from observations (black) and ERA5 (red). (**B**) Regime-dependent variability of cloud transmissivity $\sigma_{T}\left(x\right)$ used to parameterize the shortwave forcing contribution $D_{\text{SW}}\left(x\right)\propto {\left[F_{\text{SW},\text{clear}}\sigma_{T}\left(x\right)\right]}^{2}$. (**C**) The directly observed summer diffusion $D_{\text{obs}}\left(x\right)$ (solid black) compared with the two-term physics-based closure: an LWP-related estimate $D_{\text{LWP}}\left(x\right)$ (teal shading) and a shortwave-related estimate $D_{\text{SW}}\left(x\right)$ (pink shading), computed from the conditional variabilities $\sigma_{\text{LWP}}\left(x\right)$ and $\sigma_{T}\left(x\right)$ via Eq. 15. The gray dashed line is the closure sum $D_{\text{LWP}}+D_{\text{SW}}$, a physics-based prediction of $D_{\text{obs}}$ ($R^{2}=0.85$ [0.79 to 0.90] in JJA). (**D**) Seasonal variance decomposition across DJF, MAM, JJA, and SON. Colors denote the relative contribution of LWP-related (teal) and shortwave-related (pink) variability to the total inferred diffusion. All estimates derive from the 2004–2019 multivariate record.

This seasonal contrast implies that the winter LWP-based closure cannot be directly extended to summer without modification. In JJA, clouds have a higher conditional mean LWP at a given $F_{\text{LW},\text{net}}$ than in DJF (fig. S5A). As a result, longwave emissivity approaches saturation over a different part of the radiative-state space, causing the radiative sensitivity $S_{\text{LW}}\left(x\right)$ to decline at more negative $F_{\text{LW},\text{net}}$ than in winter (fig. S5B). We therefore applied the same LWP-based closure (Eq. 11) to the summer data using the JJA conditional structures of $S_{\text{LW}}\left(x\right)$ and $\sigma_{\text{LWP}}\left(x\right)$. This closure partially explains the reshaped summer diffusion structure, but captures only about a third of the observed variance ($R^{2}=0.33$; fig. S4B), indicating that an additional source of fast cloud-state variability contributes to D(x) in summer.

Motivated by this residual, and by the fact that subhourly JJA variability in $F_{\text{LW},\text{net}}$ is still dominated by $F_{\text{LW},\text{down}}$ rather than $F_{\text{LW},\text{up}}$ (fig. S3B), we next test whether rapid variability in a cloud optical property, besides the radiative property, could account for the remaining diffusion. In summer, the regime-conditional variability of transmitted shortwave flux, $\sigma_{\text{SW}}\left(x\right)$, provides an independent proxy for fast changes in cloud optical depth. Among the tested predictors, $\sigma_{\text{SW}}\left(x\right)$ shows the strongest covariation with the residual structure of D(x) (fig. S4B), suggesting that summer diffusion reflects both longwave sensitivity to LWP fluctuations and rapid optical-structure variability.

We characterize cloud-driven $\sigma_{\text{SW}}\left(x\right)$ using the normalized shortwave transmissivity

$$T\left(t\right)\equiv \frac{F_{\text{SW},\text{down}}\left(t\right)}{F_{\text{SW},\text{clear}}\left(t\right)}$$ (12)

which measures the fraction of clear-sky downwelling shortwave radiation transmitted under cloudy conditions, separating cloud-modulated transmission from slower variations in clear-sky solar forcing (“Seasonal diffusion decomposition” section). If the clear-sky shortwave flux varies slowly relative to the fluctuation timescale, shortwave perturbations can be approximated as $\delta F_{\text{SW}}\left(t\right)\approx F_{\text{SW},\text{clear}}\delta T\left(t\right)$. We therefore estimate the state-dependent shortwave variability as

$$\sigma_{\text{SW}}\left(x\right)\approx F_{\text{SW},\text{clear}}\sigma_{T}\left(x\right),\sigma_{T}\left(x\right)\equiv \text{std}\left[T|x\right]$$ (13)

We then parameterize the shortwave-related contribution as

$$D_{\text{SW}}\left(x\right)=k_{\text{SW}}\sigma_{\text{SW}}^{2}\left(x\right)$$ (14)

where $k_{\text{SW}}$ (hour^−1^) converts transmissivity variance (Fig. 5B) into effective diffusion. Here, $\sigma_{T}\left(x\right)$ acts as a proxy for rapid clear-sky breaks or cloud-fraction fluctuations missed by the LWP closure when longwave emissivity is saturated.

Extending the single-term LWP closure of Fig. 4C, we model the observed summer diffusion as the sum of physics-based longwave- and shortwave-related estimates

$$D_{\text{obs}}\left(x\right)\approx D_{\text{LWP}}\left(x\right)+D_{\text{SW}}\left(x\right)=k_{\text{LW}}{\left[S_{\text{LW}}\sigma_{\text{LWP}}\right]}^{2}+k_{\text{SW}}{\left[F_{\text{SW},\text{clear}}\sigma_{T}\right]}^{2}$$ (15)

The scaling coefficients $k_{\text{LW}}$ and $k_{\text{SW}}$ are fitted jointly via non-negative least squares; this two-term closure reproduces the directly estimated $D_{\text{obs}}\left(x\right)$ to $R^{2}=0.85$ in JJA (Fig. 5C). This single equation unifies both seasons: in summer, the shortwave term actively captures cloud-optical variability, while during polar night, the absence of sunlight ($k_{\text{SW}}\approx 0$) naturally reduces the closure to the LWP-only pathway.

Seasonal variance decomposition (Fig. 5D) shows that the model explains DJF variance almost entirely through the LWP pathway ($R^{2}=0.93$ [0.59 to 0.95]; $k_{\text{SW}}\approx 0$). During the sunlit season, the shortwave pathway dominates the explained variance in spring and summer, contributing 65% [54 to 68] in MAM and 58% [43 to 62] in JJA. Model skill is also high in these seasons ($R^{2}=0.94$ [0.76 to 0.96] and 0.85 [0.79 to 0.90], respectively). In SON, the shortwave contribution decreases to 48% [31 to 59], with lower overall skill ($R^{2}=0.59$ [0.37 to 0.79]). Overall, the summertime shortwave-related pathway yields a broad diffusion structure. Because this diffusion is broadly distributed, magnitude errors primarily rescale fluctuation intensity without introducing additional stationary modes. This explains why both ERA5 and observations retain a unimodal summer PDF despite magnitude biases. In winter, however, shortwave forcing is absent, and emissivity saturation concentrates LWP-driven variability into a sharply localized, state-dependent enhancement of D(x). Because this localized peak directly shapes the stationary structure, errors in its location or amplitude govern the presence or absence of modeled bimodality.

### Meteorological conditioning and stochastic regime transition

The preceding analysis shows that a sharply localized diffusion peak is the key dynamical ingredient sustaining winter bistability. A remaining question is what this diffusion physically represents: does it mainly reflect fast cloud-intrinsic variability, or does it also encode rapid fluctuations in the surrounding meteorological environment that project onto the radiative state? To address this, we evaluate effective potentials conditioned on background meteorology and examine how the wintertime regime structure varies with large-scale state. We address this in two steps: First, whether background meteorology reshapes the regime landscape and occupancy; and second, whether it deterministically triggers individual regime transitions.

Conditional empirical potentials are estimated from observed conditional PDFs (Fig. 6), defined as U(x∣z)≡−lnp(x∣z), where p(x∣z) is the conditional PDF of $F_{\text{LW},\text{net}}$ given the background variable *z*, $x=F_{\text{LW},\text{net}}$, and *z* denotes either the 2-m air temperature $T_{2m}$ or the surface pressure $P_{\text{sfc}}$. Wintertime conditional landscapes show that both $T_{2m}$ and $P_{\text{sfc}}$ modulate the relative depths, and hence the occupancy, of the radiative regimes, consistent with synoptic regime preference suggested in previous studies (*6*, *7*). Specifically, the conditional landscape appears as a clear-sky-dominated single-well below $T_{2m}\approx -30^{\circ }C$, a balanced double-well over the intermediate range, and a cloudy-dominated single well above $T_{2m}\approx -21^{\circ }C$ (Fig. 6A). A similar transition appears across surface pressure: The opaque cloudy well is present at all pressures, whereas the clear well becomes prominent from approximately 1007 hPa upward (Fig. 6B).

**Fig. 6.**
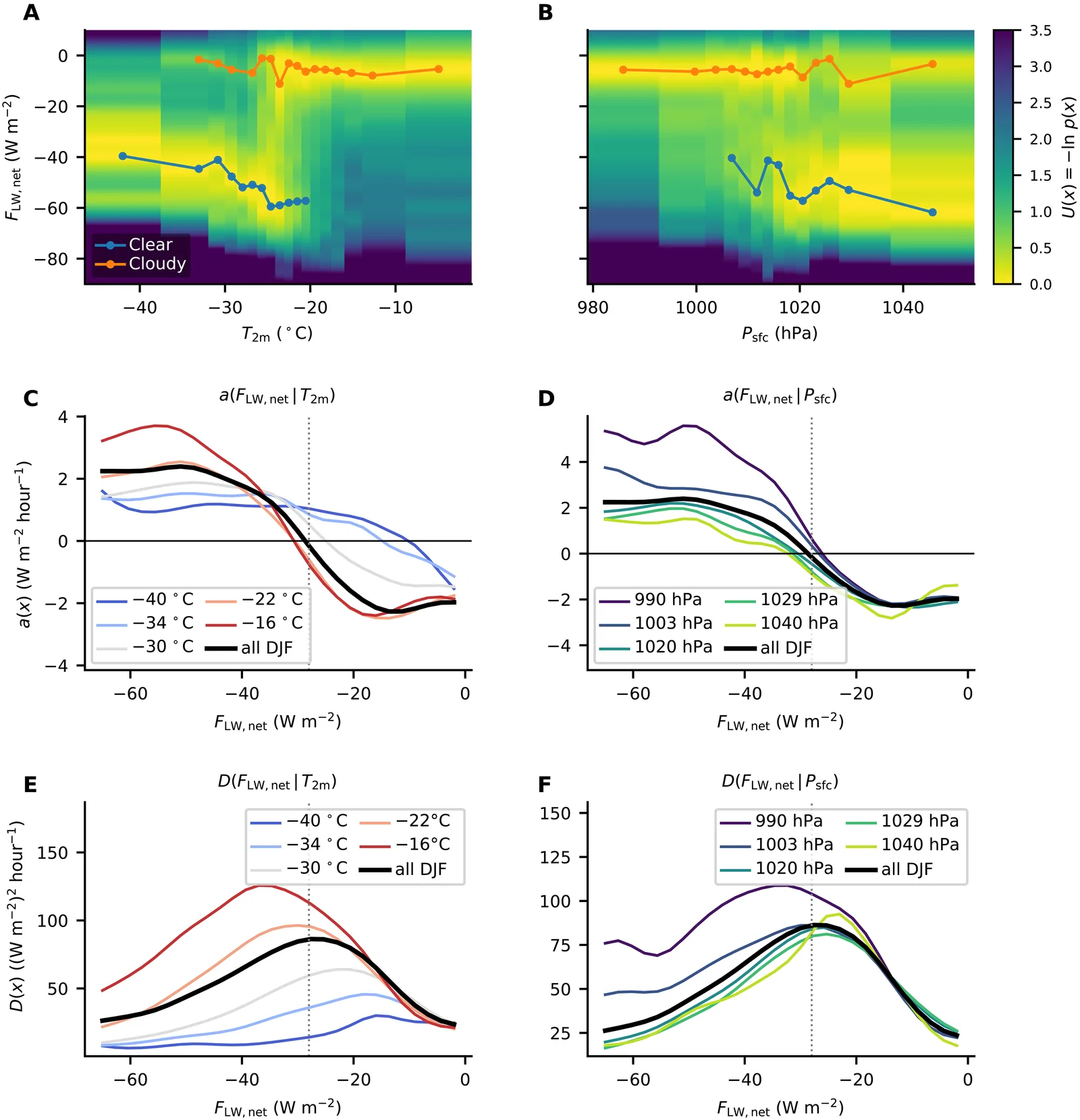
Conditional potential landscapes, drift, and diffusion under background meteorology. Conditional empirical potentials $U\left(F_{\text{LW},\text{net}}|z\right)\equiv -\text{ln}p\left(F_{\text{LW},\text{net}}|z\right)$ for (**A**) $z=T_{2m}$ and (**B**) $z=P_{\text{sfc}}$, estimated from 27 winters (DJF) of 10-min observations; blue and orange lines trace the clear and cloudy minima. (**C** and **D**) Conditional drift $a\left(F_{\text{LW},\text{net}}|z\right)$ and (**E** and **F**) conditional diffusion $D\left(F_{\text{LW},\text{net}}|z\right)$ at fixed $T_{2m}$ [(C) and (E)] and $P_{\text{sfc}}$ [(D) and (F)]; the unconditional DJF estimate is shown in black, which for both *a* and *D* matches the occurrence-frequency-weighted average of the conditional profiles; the dotted line marks the barrier ($F_{\text{LW},\text{net}}$ = −28 W m^−2^). Line colors denote the temperature and pressure bins.

These changes primarily reflect shifts in regime occupancy rather than the creation of new radiative regimes. The cloudy and clear wells remain approximately fixed in radiative space (Fig. 6), while the probability assigned to each regime changes with the meteorological condition. For example, the clear-regime fraction (time with $F_{\text{LW},\text{net}}<-28Wm^{-2}$) increases from 38% [34 to 42] below 1010 hPa to 63% [58 to 68] above 1020 hPa (fig. S6B). Similarly, clear-regime occupancy is largest at colder temperatures and gradually decreases as temperature rises, whereas cloudy-regime occupancy increases with temperature (fig. S6A). The conditional Kramers-Moyal estimates reinforce this interpretation. Under each temperature and pressure condition, the drift retains only one stable zero crossing (Fig. 6, C and D), while the diffusion retains a transition-range peak in conditions where both regimes coexist (Fig. 6, E and F). Thus, meteorological conditioning does not introduce a second deterministic equilibrium through the drift. Instead, the separation between the clear and cloudy regimes remains associated with state-dependent diffusion in the transition range.

Within this occupancy-shift picture, pressure and temperature affect the two-regime structure in different ways. For pressure, both $a\left(x|P_{\text{sfc}}\right)$ and $D\left(x|P_{\text{sfc}}\right)$ remain relatively consistent across pressure conditions, apart from the lowest-pressure bin near 990 hPa. This exception does not appear to indicate a direct pressure control on D(x): At the lowest pressures, the sampled conditions are predominantly warm, suggesting that the apparent pressure dependence of D(x) arises indirectly through covariation with $T_{2m}$ rather than through pressure itself (fig. S6C). We therefore focus on interpreting the temperature dependence of $D\left(x|T_{2m}\right)$.

To test whether the temperature dependence is consistent with the cloud-liquid mechanism, we apply the temperature-conditioned LWP-based closure (Eq. 11). The closure improves systematically with temperature: It reproduces the observed $D\left(x|T_{2m}\right)$ well for $T_{2m}\geq -19^{\circ }C$ (r=0.76 [0.31 to 0.85]) and for $-25\leq T_{2m}<-19^{\circ }C$ (r=0.67 [0.15 to 0.81]), where liquid water is sufficient, but weakens for $T_{2m}<-25^{\circ }C$ (r=0.16 [−0.04 to 0.52]), where liquid water is scarce and there is little LWP variability for the closure to act on. This indicates that the temperature dependence of diffusion is largely mediated by LWP variability where liquid water is available. In this interpretation, temperature does not create the diffusion peak directly, but modulates the distribution of LWP and the shape of $S_{\text{LW}}$, which together determine how liquid-water variability is mapped onto $F_{\text{LW},\text{net}}$, as discussed for the seasonal contrast in the “Seasonal decomposition of state-dependent diffusion” section. Together, these results show that $T_{2m}$ and $P_{\text{sfc}}$ do not create the clear and cloudy regimes, but modulate their relative occupancy. The separation between regimes is maintained by state-dependent diffusion, whereas background meteorology controls how probability is assigned to each regime.

We further test whether individual regime crossings are controlled by deterministic meteorological triggers or by unresolved fast variability. If crossings were primarily driven by concurrent meteorological changes, the short-term changes in $F_{\text{LW},\text{net}}$ immediately before crossing should be predictable from observed meteorological and cloud-state variables. We therefore use a hierarchical, tree-based XGBoost regression (*27*) to decompose the variance of hourly changes $\Delta F_{\text{LW},\text{net}}$ during the 1 to 3 hours before each barrier crossing. The analysis is performed separately by transition direction over 22 DJF seasons with consistent LWP coverage (“Regime classification and transition detection” section).

Predictors include both the instantaneous values, hereafter referred to as levels, and hourly tendencies of meteorological and cloud variables, where each tendency is computed as the one-hour change in the corresponding variable. Level-only variables yield near-zero explained variance ($R^{2}<5\%$), and standard linear regression captures only ∼20%, confirming that pretransition variability is nonlinear and relies on temporal tendencies rather than static conditions (fig. S7). The full XGBoost model, i.e., including tendencies, explains 33.1% of pretransition $\Delta F_{\text{LW},\text{net}}$ variance before clear-to-cloudy transitions and 33.9% before cloudy-to-clear transitions (Fig. 7, A and B). The hierarchy shows that surface and upper-level meteorological variables together explain only ∼10% of the variance. Adding surface turbulent fluxes increases the explained variance to ∼15 to 16%, while LWP and cloud-base-property tendencies provide the dominant additional contribution, accounting for another ∼17 percentage points in both transition directions. Because $F_{\text{LW},\text{net}}$ responds directly to LWP through cloud emissivity, this added skill is expected and does not, by itself, identify a deterministic meteorological trigger. Rather, LWP acts as an observed reduced-order descriptor of rapidly varying cloud-liquid conditions that project onto $F_{\text{LW},\text{net}}$ through the liquid-water-path–emissivity–longwave coupling. The substantive result is therefore the weak predictive skill of the resolved environment: Surface and upper-level meteorology, even when hourly tendencies are included, explain only a small fraction of the pretransition variance.

**Fig. 7.**
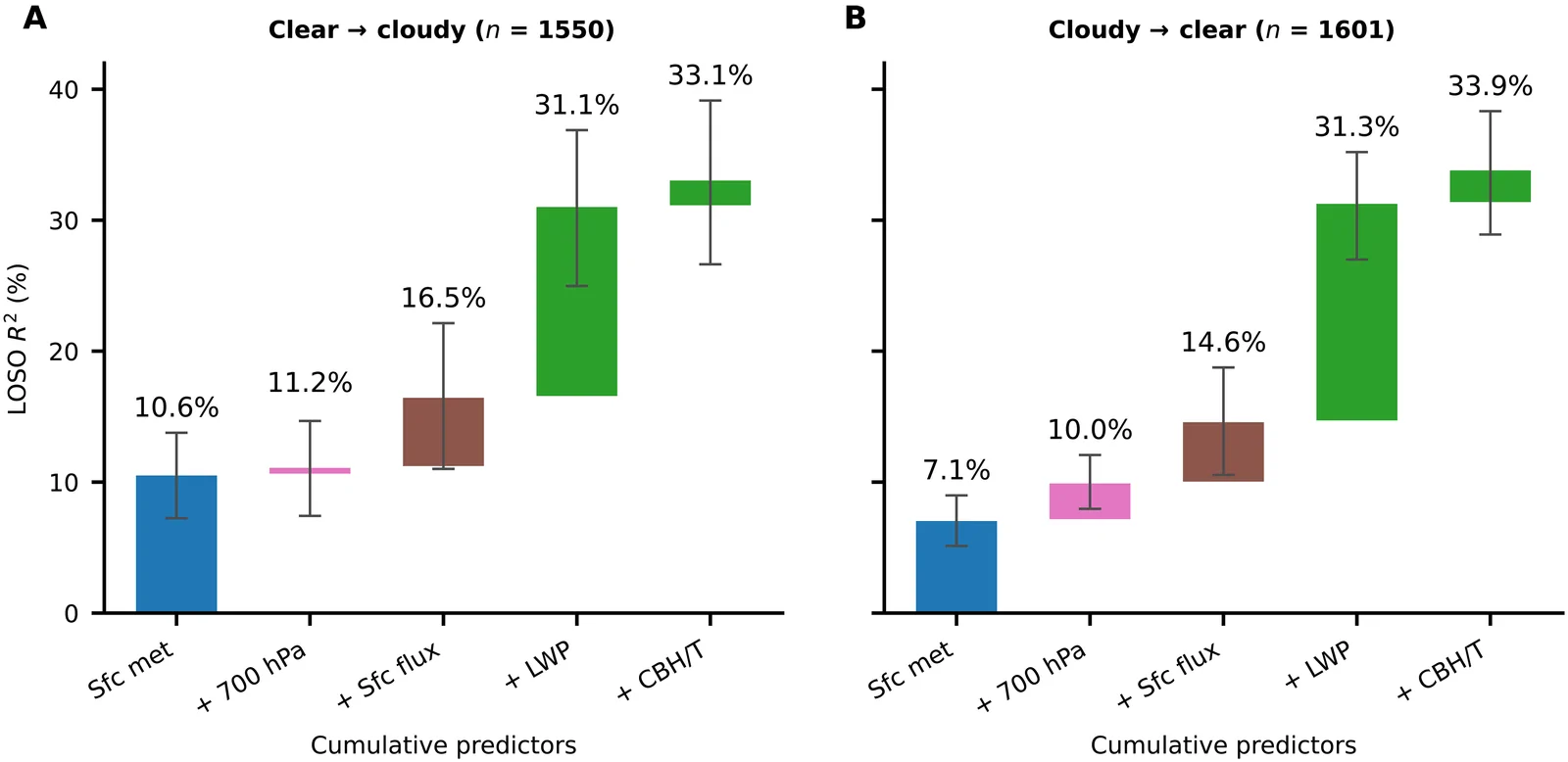
Meteorological covariability during regime transitions. Hierarchical variance decomposition of hourly $\Delta F_{\text{LW},\text{net}}$ via leave-one-season-out XGBoost regression (cumulative $R^{2}$, %) for the 1 to 3 hours preceding (**A**) clear-to-cloudy (n=1550) and (**B**) cloudy-to-clear (n=1601) barrier crossings across 22 DJF seasons. Each bar adds a variable group using both state and hourly tendency features. Colors indicate variable categories: surface meteorology (temperature, wind direction/velocity, pressure, relative humidity; blue), 700 hPa reanalysis (temperature *T*, specific humidity *q*, vertical velocity ω, and precipitable water; pink), surface turbulent fluxes (sensible and latent heat; brown), LWP, and cloud-base height/temperature (green). Error bars show 95% bootstrap confidence intervals on the mean seasonal $R^{2}$.

The remaining Utqiaġvik variance could partly reflect missing or imperfectly observed predictors rather than irreducible stochasticity. To test this, we apply the same framework to MOSAiC central-Arctic observations (DJF 2019–2020; fig. S7, B and D), where continuous ice water path retrievals and colocated eddy-covariance surface fluxes are available. The explained variance is substantially higher: 42% [36 to 48] for clear-to-cloudy and 56% [48 to 63] for cloudy-to-clear transitions. At MOSAiC, adding measured surface turbulent fluxes and ice water path further improves explained variance, with turbulent fluxes dominating the added gain. Adding these fluxes raises explained variance from 5.3% [−0.6 to 10.7] to 31.3% [21.1 to 41.2] for clear-to-cloudy transitions and from 14.5% [0.6 to 28.4] to 49.5% [36.7 to 59.4] for cloudy-to-clear transitions, compared with only ∼5% at Utqiaġvik, where fluxes were estimated using bulk aerodynamic formulas. This comparison suggests that part of the unexplained variance at Utqiaġvik reflects missing or imperfectly observed predictors. However, even with richer colocated observations, concurrent meteorology and cloud properties explain at most ∼56% of the pretransition radiative variability. The residual therefore argues against a single consistent meteorological trigger captured by the observed environment. Resolved meteorology appears to set the slow envelope of regime occupancy, whereas event-scale crossings retain a substantial component that is not predicted by concurrent observed predictors, consistent with fast stochastic variability during regime transitions.

## DISCUSSION

The key to understanding Arctic winter radiative bistability lies in the transition range ($F_{\text{LW},\text{net}}\approx -40$ to $-10Wm^{-2}$), where subhourly cloud-driven variability produces a localized enhancement of the diffusion coefficient D(x) that separates the clear and opaque regimes, even though the drift has only a single equilibrium (Fig. 8D). Thus, the observed two-regime structure does not require deterministic multistability. Instead, fast cloud-driven variability serves to maintain bimodal regime structure in the reduced stochastic dynamics by shaping the state-dependent diffusion. This interpretation is supported by three independent Arctic observing records from Utqiaġvik, Ny-Ålesund, and MOSAiC. It also explains the persistent bias of reanalyses such as ERA5 toward a single intermediate state: the failure to sustain distinct regimes arises primarily from underrepresented state-dependent cloud variability rather than from bias in the large-scale mean restoring tendency alone.

**Fig. 8.**
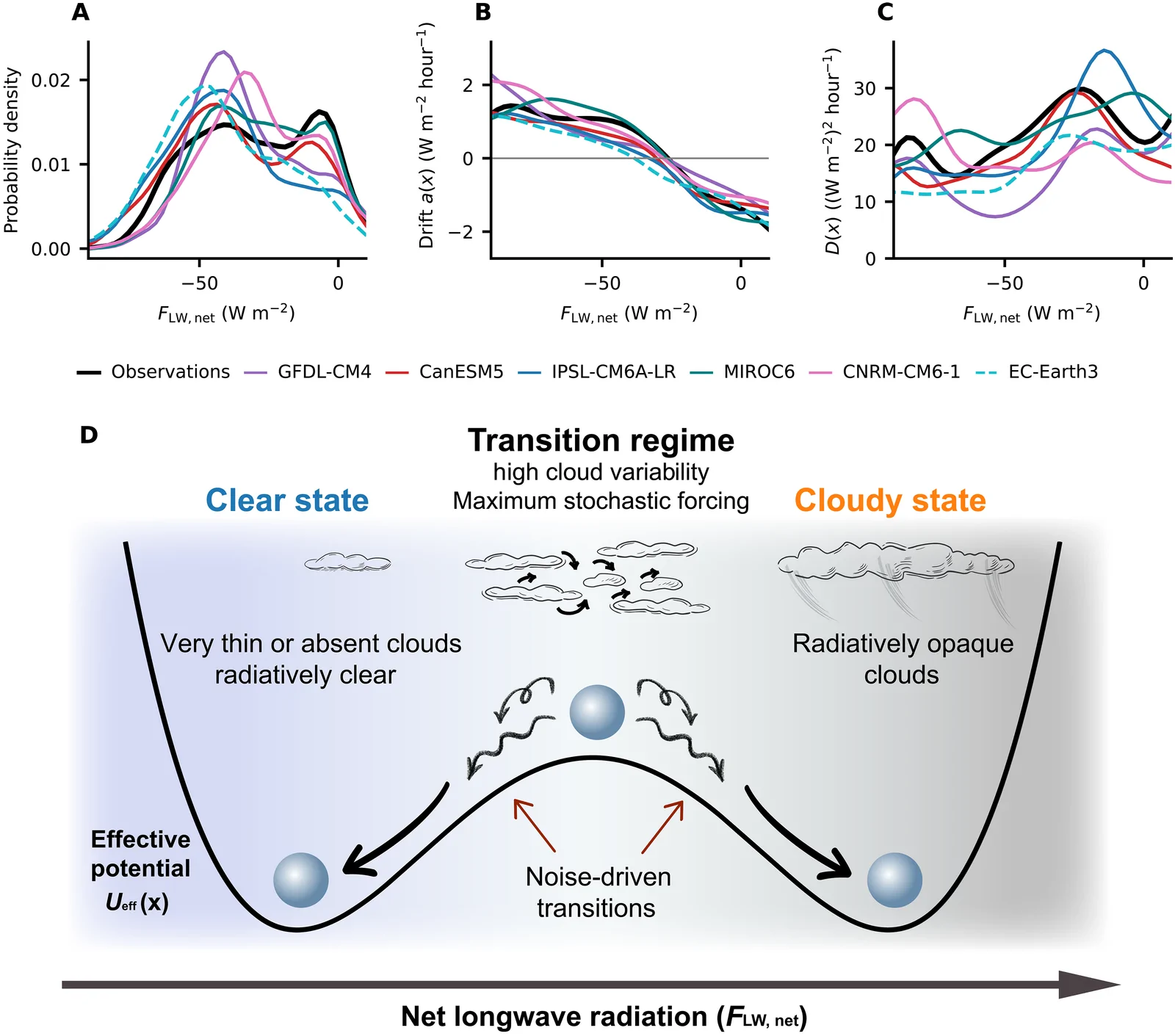
Noise-induced bistability in Arctic winter radiative regimes. (**A**) Probability density, (**B**) effective drift a(x), and (**C**) state-dependent diffusion D(x) for observations (black) and six CMIP6 models (colored lines) at 3-hourly resolution (DJF 1998/1999–2013/2014). (**D**) Schematic illustrating how state-dependent stochastic forcing produces bimodal regimes in surface net longwave radiation. The effective potential $U_{\text{eff}}\left(x\right)$ as a function of net longwave radiation $F_{\text{LW},\text{net}}$ contains two stable wells corresponding to a clear-sky radiative state (left) and a cloudy radiative state (right). The clear state is characterized by thin or absent clouds and vanishingly low LWP, while the cloudy state corresponds to a thicker stratiform cloud layer with higher LWP. Between these regimes lies a transition region where cloud properties are highly variable. As a result, the system state (ball) undergoes random, noise-driven excursions that occasionally cross the barrier separating the two wells, producing intermittent transitions between clear and cloudy radiative regimes.

These results further indicate that the observed bimodality cannot be attributed solely to alternating synoptic states (*6*). Conditional potential landscapes show that background meteorology modulates the relative occupancy of the clear and cloudy regimes, but does not create distinct drift equilibria within the reduced one-dimensional dynamics. Across the meteorological conditions examined, the inferred drift retains a single-equilibrium restoring structure. Thus, meteorology shifts the relative occupancy of the two regimes, whereas the regime separation itself is maintained by the localized enhancement of state-dependent diffusion in the transition range. Although hidden-state or nonstationary influences cannot be excluded, the weak predictive power of concurrent meteorological variables for imminent transitions indicates that large-scale forcing alone is insufficient to explain the observed switching. A natural extension is a multidimensional SDE framework that includes external variables such as temperature and pressure, which could quantify how meteorological conditions modulate regime occupancy and reshape the effective potential.

The wintertime diffusion peak originates from nonlinear coupling between cloud-liquid variability and longwave radiative sensitivity. Physically, it reflects two competing tendencies. LWP variability increases with cloud thickness, whereas radiative sensitivity decreases as emissivity saturates. The product of these two factors peaks in the transition range, where clouds remain thin enough for a strong emissivity response yet hold enough liquid water for subhourly LWP fluctuations to be radiatively amplified. This explains why the diffusion coefficient peaks between the clear and cloudy regimes rather than within either regime. More broadly, these LWP fluctuations provide an observable projection of multiple unresolved fast processes, including transient changes in boundary-layer turbulence, surface heat fluxes, and local microphysics.

The Coupled Model Intercomparison Project Phase 6 (CMIP6) model ensemble provides a broader test of whether the diffusion-peak requirement extends beyond ERA5. Across six CMIP6 models (*28*) analyzed at matched 3-hourly resolution (Fig. 8), the intermodel spread in winter radiative bimodality is organized by the diffusion structure rather than by the effective drift: The inferred drift resembles observations in all models and cannot explain the spread (Fig. 8B), whereas models that reproduce a transition-range peak in D(x), most clearly CanESM5, also reproduce the bimodal $F_{\text{LW},\text{net}}$ PDF. Models with a weak or displaced peak, such as MIROC6, remain weakly bimodal or effectively unimodal (Fig. 8, A and C). Horizontal resolution does not control this spread: CanESM5 captures the bimodality despite having the coarsest grid (∼2.8°), whereas ERA5 remains effectively unimodal at 0.25° (Fig. 1C).

In our framework, the diffusion peak depends on the product of longwave radiative sensitivity and LWP variability. This relationship suggests that model spread in winter bimodality is tied to how models represent supercooled liquid variability. Schemes that deplete liquid too rapidly through glaciation (*16*) would suppress the LWP fluctuations needed to produce the transition-range diffusion peak. The two models with subdaily cloud-liquid output support this interpretation. In ERA5, $S_{\text{LW}}\left(x\right)$ is broadly consistent with observations, but fast LWP fluctuations are underestimated, especially in the clear and transition regimes (fig. S8, A and B). CanESM5 reproduces the bimodal PDF because it retains a localized transition-range peak in D(x). To diagnose the cloud-liquid pathway, we use the corresponding CanESM5 Atmospheric Model Intercomparison Project (AMIP) output, which was run with the same cloud microphysics scheme as the historical simulation (*29*) and provides 3-hourly cloud-water fields. We find that its LWP variability agrees best near the transition range, while biases in both $S_{\text{LW}}\left(x\right)$ and $\sigma_{\text{LWP}}\left(x\right)$ in the clear and cloudy regimes offset each other (fig. S8, A and B). The model comparison therefore identifies the key requirement: a localized transition-range enhancement of D(x). Because subdaily LWP output is unavailable for most CMIP6 models, subdaily cloud-liquid variability should be a practical evaluation target for reducing Arctic winter mixed-phase cloud and surface-radiation biases.

The stochastic model used here should be interpreted as an effective reduced description inferred from nonparametric Kramers-Moyal estimates, not as an exact closed-form representation of the full cloud-radiation system. The leading-order SDE retains only the first two conditional increment moments and therefore neglects possible higher-order dependencies. Sensitivity tests show that these higher-order terms do not alter the main conclusion (“Kramers-Moyal estimation of drift and diffusion” section). Strict stationarity over the full multidecadal record is also not assumed. Instead, we interpret the DJF process as quasi-stationary: Slow interannual changes may shift regime occupancy (fig. S9C), while the short-time drift and diffusion structure remains persistent over the analysis timescale.

This interpretation shifts the climate implication from the mean radiative state to the variability structure that controls regime occupancy. During Arctic winter, the residence time of the boundary layer in clear and cloudy radiative regimes regulates surface longwave cooling, thermodynamic sea-ice growth, and surface–boundary-layer coupling. A model may therefore reproduce the mean wintertime longwave flux while still misrepresenting Arctic surface energy exchange if it collapses the observed bimodal regime structure into a single intermediate state. This provides a concrete target for model evaluation. Models should be tested not only against mean radiative fluxes, but also against the observed state-dependent diffusion structure, including the shape of D(x), and the resulting regime persistence. Stochastic parameterizations offer one route for representing this unresolved, state-dependent variability in climate models (*30*–*32*). At the same time, coordinated observations combining radiative fluxes, ice-nucleating particles, cloud microphysics, and surface turbulent fluxes are needed to determine how much of the inferred diffusion reflects identifiable fast physical processes rather than irreducible stochastic forcing.

In conclusion, we show that a climate-relevant radiative regime structure as represented by the bimodal Arctic $F_{\text{LW},\text{net}}$ PDF is maintained by state-dependent fast variability rather than by a mean tendency. Because climate change will alter not only the mean atmospheric state but also the structure of cloud-related variability around it, future changes in Arctic winter radiative regimes may depend as much on shifts in the variability landscape as on shifts in the mean atmospheric state.

## MATERIALS AND METHODS

### Observational, reanalysis, and model datasets

We analyze surface longwave radiative fluxes at Utqiaġvik, Alaska (71.3°N, 156.6°W) using the 1-min resolution (1998–2025) station record from the NOAA Global Monitoring Laboratory (GML) Baseline Surface Radiation Network (BSRN), which is colocated with the U.S. Department of Energy Atmospheric Radiation Measurement (ARM) North Slope of Alaska (NSA) facility. For SDE estimation, we resample the 1-min data to 10-min means. We apply identical preprocessing to all seasonal subsets (DJF, MAM, JJA, and SON) drawn from this record. Samples outside the physical range $-150\leq F_{\text{LW},\text{net}}\leq 50Wm^{-2}$ are removed from all seasonal subsets. Such excursions are absent in DJF, rare in MAM and JJA (0.02%, 0.1% of the 1-min data), and concentrated in SON (0.5%, reaching $|F_{\text{LW},\text{net}}|\approx 2\times 10^{4}Wm^{-2}$).

We use two complementary LWP retrievals at the colocated ARM NSA site, chosen to match the requirements of each analysis. For the physics-based diffusion closure (“Radiative sensitivity and LWP variability explain the peaked diffusion” section), which depends on the shape of the conditional variability profile $\sigma_{\text{LWP}}\left(x\right)$, we use the multisensor Shupe-Turner retrieval (*33*), which combines radar, lidar, and microwave radiometer observations to provide accurate liquid-phase discrimination (2004–2019). For the variance decomposition (“Hierarchical variance decomposition”), we use the ARM microwave-radiometer (MWR) best-estimate LWP, which provides *22* DJF seasons of continuous coverage (2001–2023) and maximizes the leave-one-season-out cross-validation sample. Both radiation and LWP records are resampled to 10-min means.

We extract ERA5 hourly surface longwave fluxes (*15*) from the nearest grid point to the observation site (71.25°N, 156.75°W; 0.25° native resolution) and map them to the same net longwave state definition. We analyze ERA5 at its native hourly resolution, applying identical diagnostics (PDFs, drift, and diffusion inference) to both observations and ERA5 at their respective sampling intervals. Because the inferred SDE coefficients depend on the estimation lag Δt, we compare observations (10-min) and ERA5 (1-hour) primarily for qualitative structure; we assess sensitivity to Δt in the “SDE model validation” section.

To test whether coupled climate models capture the observed bistability, we analyze 3-hourly surface net longwave fluxes from six CMIP6 historical simulations extracted at the nearest grid point to Utqiaġvik: GFDL-CM4, CanESM5, IPSL-CM6A-LR, MIROC6, CNRM-CM6-1, and EC-Earth3 (*28*). For direct comparability with the model output, we resample the observations to 3-hourly means. We restrict all datasets to DJF 1998/1999–2013/2014 to ensure a consistent historical comparison (Fig. 8). For the cross-source closure decomposition (fig. S8), we additionally use 3-hourly cloud-water output from the CanESM5 AMIP simulation, together with ERA5 total-column cloud liquid water; all records are resampled to a common 3-hourly cadence before computing conditional statistics.

To assess multisite representativeness, we apply the same inference pipeline to two independent surface-radiation records: the BSRN station at Ny-Ålesund, Svalbard (79°N; 1-min resolution; 2006–2025) (*34*), and surface radiation measurements from the central-Arctic MOSAiC drift expedition (85°N; 10-min native cadence; DJF 2019–2020) (*23*) (fig. S1).

### Stochastic inference framework

#### Kramers-Moyal estimation of drift and diffusion

We estimate the drift and diffusion of Eq. 1 using a nonparametric Kramers-Moyal approach based on finite-lag conditional moments evaluated at the sampling interval τ=Δt (*21*, *26*). For increments $\Delta x_{n}\equiv x_{n+1}-x_{n}$ over $\Delta t_{n}\equiv t_{n+1}-t_{n}$, the estimators at grid points $x_{g}$ are

$$\hat{a}\left(x_{g}\right)=\frac{\Sigma_{n}w_{n}\left(x_{g};h\right)\Delta x_{n}}{\Sigma_{n}w_{n}\left(x_{g};h\right)\Delta t_{n}}$$ (16)

$$\hat{D}\left(x_{g}\right)=\frac{1}{2}\frac{\Sigma_{n}w_{n}\left(x_{g};h\right)\Delta x_{n}^{2}}{\Sigma_{n}w_{n}\left(x_{g};h\right)\Delta t_{n}}$$ (17)

corresponding to kernel-weighted estimates of 〈Δx∣x〉/Δt and $\frac{1}{2}\langle \Delta x^{2}|x\rangle /\Delta t$. We interpret these finite-lag estimators as effective Itô coefficients of the reduced process, consistent with Eq. 1. Accordingly, when diffusion is state-dependent, the inferred drift $\hat{a}\left(x\right)$ should be interpreted as an effective drift, encompassing both the underlying deterministic mean tendency and any noise-induced contribution associated with the state dependence of $\hat{D}\left(x\right)$ (*19*).

Kramers-Moyal coefficients are estimated using the Nadaraya-Watson kernel regression approach (*35*) with a Gaussian kernel in the state $x_{n}$

$$w_{n}\left(x_{g};h\right)=\text{exp}\left[-\frac{{\left(x_{n}-x_{g}\right)}^{2}}{2h^{2}}\right]$$ (18)

Unless stated otherwise, we use a fixed bandwidth $h=2Wm^{-2}$ and evaluate $\hat{a}\left(x\right)$ and $\hat{D}\left(x\right)$ on a uniform *x*-grid. To reduce sampling noise, we smooth the inferred curves on the *x*-grid using a Gaussian filter with SD $\sigma_{s}=2\Delta x$ (where Δx is the grid spacing). We apply this smoothing solely to suppress sampling noise in the bin-wise estimates; all reported results remain robust to reasonable changes in the smoothing width (“SDE model validation” section).

This nonparametric reconstruction presumes that the reduced process is approximately Markovian, time-homogeneous, and densely sampled, with a sampling interval short compared with the dynamical relaxation time (*22*, *36*). We verify each condition to a reasonable degree: Markov consistency through the Chapman-Kolmogorov test (“SDE model validation” section; fig. S2D), approximate time-homogeneity through the quasi-stationary analysis (moving-window, early/late-split, and detrending tests; “SDE model validation” section), dense sampling through the multidecadal 10-min record, and the short-lag condition through the estimation-lag sweep (Δt = 10 to 60 min; fig. S2, A to C), with Δt much shorter than the multihour drift-relaxation and regime-residence timescales.

The second-order Kramers-Moyal truncation is used as an effective leading-order drift-diffusion closure, not an assumption of Gaussian finite-lag increments. By the Pawula theorem, such a truncation is exact only when the higher-order coefficients vanish (*37*). The short-lag increments are leptokurtic: the normalized increment kurtosis $Q\left(\tau \right)\equiv M_{4}/\left(3M_{2}^{2}\right)$, where $M_{2}$ and $M_{4}$ are the second (variance) and fourth central moments of the lag-τ increments (so that Q=1 for a Gaussian), is Q=7.8 [6.8 to 8.8] at τ = 10 min, decreasing to 3.3 [3.0 to 3.7] at τ = 60 min under temporal aggregation. This argues against scale-invariant infinite-variance α-stable Lévy forcing, for which the fourth moment is not finite (*38*), and supports a finite-variance interpretation compatible with jump-like contributions (*36*, *39*). As a sensitivity check following (*39*), we represented the unresolved fast variability as an admissible compound-Poisson jump process, conservatively assigning the measured fourth-moment rate entirely to the jump term (a near-maximal jump contribution). Nevertheless, the reconstructed stationary density changes by only 4.9% [3.6 to 6.7] in total-variation distance, leaving the bimodality and the transition-range diffusion peak intact (fig. S10). We therefore treat the inferred Kramers-Moyal coefficients as effective quantities that may include finite-variance discontinuous contributions.

Conditional drift and diffusion (Fig. 6, C to F) use the same finite-lag Kramers-Moyal estimator (Eqs. 16 to 17), extended with an additional Gaussian kernel in the conditioning variable *z*. For each state grid point $x_{g}$ and conditioning value $z_{c}$, the weight is the product $w_{n}\left(x_{g},z_{c}\right)=\text{exp}\left[-{\left(x_{n}-x_{g}\right)}^{2}/2h_{x}^{2}\right]\text{exp}\left[-{\left(z_{n}-z_{c}\right)}^{2}/2h_{z}^{2}\right]$, and the coefficients follow as $\hat{a}\left(x_{g}|z_{c}\right)=\sum_{n}w_{n}\Delta x_{n}/\sum_{n}w_{n}\Delta t_{n}$ and $\hat{D}\left(x_{g}|z_{c}\right)=\frac{1}{2}\sum_{n}w_{n}\Delta x_{n}^{2}/\sum_{n}w_{n}\Delta t_{n}$. We use a state bandwidth $h_{x}=4Wm^{-2}$ (wider than in the unconditional estimate to compensate for the smaller per-condition samples) and conditioning bandwidths $h_{z}$ = 3°C for $T_{2m}$ and 5 hPa for $P_{\text{sfc}}$, evaluated at $T_{2m}\in {\left\{-40,-34,-30,-22,-16\right\}}^{\circ }C$ and $P_{\text{sfc}}\in \{990,1003,1020$, 1029, 1040} hPa. Grid points with summed kernel weight below 60 are discarded to avoid unstable estimates in sparsely sampled bins, and kernel statistics are accumulated per winter to support winter-block bootstrap confidence intervals.

*Parametric deformation of the diffusion profile.* To construct Fig. 3A, we define a one-parameter family of diffusion profiles, $D_{\alpha }\left(x\right)$, while keeping the effective drift a(x) fixed to the observational estimate shown in Fig. 2A. Let $D_{\text{flat}}$ denote the nearly flat ERA5-based reference diffusion profile and $D_{\text{obs}}\left(x\right)$ the observationally inferred diffusion profile. We define α=1 as the flat reference case and $\alpha =\alpha_{\text{obs}}$ as the observed case.

For $1\leq \alpha \leq \alpha_{\text{obs}}$, the diffusion profile is interpolated as

$$D_{\alpha }\left(x\right)=\left[1-\lambda \left(\alpha \right)\right]D_{\text{flat}}+\lambda \left(\alpha \right)D_{\text{obs}}\left(x\right),\lambda \left(\alpha \right)=\frac{\alpha -1}{\alpha_{\text{obs}}-1}$$ (19)

so that $D_{\alpha =1}\left(x\right)=D_{\text{flat}}$ and $D_{\alpha =\alpha_{\text{obs}}}\left(x\right)=D_{\text{obs}}\left(x\right)$.

For $\alpha >\alpha_{\text{obs}}$, the observed shape is held fixed and only the overall amplitude is increased

$$D_{\alpha }\left(x\right)={\left(\frac{\alpha }{\alpha_{\text{obs}}}\right)}^{2}D_{\text{obs}}\left(x\right)$$ (20)

The quadratic factor is used because α is defined as a noise-amplitude parameter, whereas diffusion enters the SDE as a variance-like quantity.

As a control, we also consider uniform amplification of the flat reference diffusion without introducing state dependence,

$$D_{\alpha }^{\text{ctrl}}\left(x\right)=\alpha^{2}D_{\text{flat}}$$ (21)

For each prescribed diffusion profile, the corresponding stationary density is computed using the same one-dimensional SDE framework as in the main analysis.

#### SDE model validation

We validate the inferred one-dimensional SDE using the following complementary checks.

*Stationarity.* The stationary Fokker-Planck reconstruction is an effective DJF-conditioned representation of the reduced process, not a claim that the full multidecadal record is strictly stationary. The requirement is weaker: The short-time drift–diffusion structure need only remain approximately stable, while slow climate variability may shift the relative occupation of the clear and cloudy regimes. This separation is large here: The relaxation time $\tau_{\text{drift}}\approx 6.7\text{hours}$ (Fig. 2) is far shorter than a DJF season (∼2160 hours) and interannual-to-decadal variability, so each winter samples many relaxation times.

Formal tests support this interpretation. For the pooled multidecadal record, the augmented Dickey-Fuller tests [ADF and ADF-GLS (*40*)] reject a unit root (P<0.001 across all deterministic specifications and lags 1 to 144), while the Kwiatkowski-Phillips-Schmidt-Shin (KPSS) statistic (*41*) rejects strict level-stationarity (1.6 and 2.5 at hourly and 10-min resolution; 1% critical value 0.74). Together these indicate a slow, long-term trend in the mean level rather than a stochastic trend. Resolved within winters, the linearly detrended series are stationary (ADF and ADF-GLS reject the unit root in 27/27 winters; KPSS does not reject stationarity in any) and re-estimating the coefficients on the detrended series leaves a(x) and D(x) essentially unchanged (pattern correlation r≥0.99).

We confirm that the inferred dynamics are robust to this slow trend using moving-window Kramers-Moyal estimates (*42*) and an independent early/late split. Across sliding seven-winter windows, the inferred drift remains monostable (fig. S9A), the diffusion enhancement stays concentrated in the clear–cloudy transition range (fig. S9B), and the window-wise empirical PDF remains bimodal (fig. S9C). The early/late split agrees: coefficients inferred from 1998–2010 generalize to 2011–2025 (pattern correlations r>0.98 for both a(x) and D(x), reproducing the effective well structure and regime residence statistics through Euler-Maruyama integration (fig. S11). Together, these tests support a quasi-stationary process, stationary around a slowly varying long-term mean, in which the drift-diffusion structure is trend-insensitive and reflects within-winter dynamics, while the slow change in relative well occupation carries the low-frequency warming signal (fig. S9C).

*Markov consistency*. We further assess finite-lag sensitivity by repeating the Kramers-Moyal reconstruction over a range of estimation lags Δt, verifying that the inferred coefficients [$\hat{a}\left(x\right)$ and $\hat{D}\left(x\right)$] and the resulting stationary PDF vary only weakly across the tested range (fig. S2, A to C). Last, we assess consistency with the Markov assumption underlying the effective one-dimensional SDE using the Chapman-Kolmogorov equation (*22*, *43*): Transition densities reconstructed from successive Δt transitions closely reproduce the directly estimated 2Δt transition densities (pattern correlation r≥0.98 at 2Δt and r>0.91 out to 5Δt for both 10-min and 1-hour base lags; fig. S2D). The gradual decrease in correlation at longer prediction horizons indicates residual memory from unresolved processes.

*Necessity of state-dependent diffusion*. A bimodal stationary PDF alone does not uniquely identify the underlying stochastic dynamics, because an additive-noise process can be constructed to reproduce a given stationary distribution by modifying the drift. To make this nonuniqueness explicit, we replaced the inferred state-dependent diffusion coefficient D(x) by a constant $D_{0}=\langle D\rangle$ and inferred the corresponding drift $\tilde{a}\left(x\right)$ from the stationary Fokker-Planckrelation so that the additive model reproduces the same stationary PDF, $p_{\text{st}}\left(x\right)$ (*26*). This PDF-matched additive model and the inferred state-dependent model therefore yield identical stationary distributions by construction (fig. S12) (*19*).

The support for state-dependent diffusion instead comes from the independently estimated dynamics: (i) the Kramers-Moyal estimate directly gives a strongly state-dependent *D*(*x*), varying by a factor of 4.1 across the observed states (Fig. 2B), and (ii) this state dependence is independently explained by the physical closure $D\propto {\left[S_{\text{LW}}\sigma_{\text{LWP}}\right]}^{2}$ (Fig. 4). Thus, while an additive-noise drift can be tuned to reproduce the same stationary PDF, it does not reproduce the inferred diffusion structure or its physical interpretation.

### Physics-based interpretation of diffusion

#### Longwave radiative sensitivity

We model the downwelling longwave flux beneath an Arctic cloud layer using a Beer-Lambert emissivity,

$$\epsilon \left(\text{LWP}\right)=1-e^{-\kappa_{\text{eff}}\text{LWP}}$$ (22)

so that the downwelling longwave flux is

$$F_{\text{LW},\text{down}}\left(\text{LWP}\right)\approx F_{\text{clear}}+F_{\text{max}}\epsilon \left(\text{LWP}\right)$$ (23)

where $F_{\text{clear}}$ is the clear-sky downwelling flux, and $F_{\text{cloudy}}^{\text{bb}}$ is its opaque-cloud (blackbody, ϵ=1) counterpart, so that $F_{\text{max}}\equiv F_{\text{cloudy}}^{\text{bb}}-F_{\text{clear}}$ is the maximum cloud longwave enhancement; $\kappa_{\text{eff}}$ is the effective broadband absorption scale for LWP. Because $F_{\text{LW},\text{up}}$ (controlled by surface temperature) varies slowly compared with LWP on the subhourly timescales used to infer D(x), the downwelling component dominates the instantaneous sensitivity (fig. S3A) of $x\equiv F_{\text{LW},\text{net}}$ to LWP

$$\frac{\partial x}{\partial \text{LWP}}\approx \frac{\partial F_{\text{LW},\text{down}}}{\partial \text{LWP}}$$ (24)

Differentiating yields the longwave radiative sensitivity

$$S_{\text{LW}}\left(\text{LWP}\right)\equiv \frac{\partial x}{\partial \text{LWP}}\approx F_{\text{max}}\kappa_{\text{eff}}e^{-\kappa_{\text{eff}}\text{LWP}}$$ (25)

which decreases exponentially with LWP. Thin clouds remain radiatively sensitive to additional liquid, whereas thicker clouds approach blackbody-like emission (ϵ→1) and become largely insensitive.

The Beer-Lambert emissivity form is a standard approximation in longwave cloud optics and General Circulation Model radiation schemes (*44*–*46*). Previous studies found mass absorption coefficients to range from 0.09 to 0.16 (g m^−2^)^−1^ depending on the spectral band, the convention for the diffusivity factor (*45*), and whether the target is cloud emissivity or surface flux (*47*, *48*). Because our single-exponential model maps LWP directly to the broadband surface downwelling longwave flux rather than to cloud emissivity alone, we interpret $\kappa_{\text{eff}}$ as an effective broadband surface absorption scale that absorbs spectral weighting, atmospheric window contributions, and cloud-base temperature effects.

We independently diagnose the transfer-function parameters from the observed $F_{\text{LW},\text{down}}$-LWP relationship at Utqiaġvik. We constrain $F_{\text{clear}}$ using observed clear-sky downwelling longwave fluxes and fit $F_{\text{max}}$ and $\kappa_{\text{eff}}$ via nonlinear least squares. The resulting values are $\kappa_{\text{eff}}=0.045{\left(gm^{-2}\right)}^{-1}$, $F_{\text{max}}=83.8Wm^{-2}$, and $F_{\text{clear}}\approx 171Wm^{-2}$ (hence, $F_{\text{cloudy}}^{\text{bb}}\approx 255Wm^{-2}$). The flux parameters are physically plausible: $F_{\text{clear}}\approx 171Wm^{-2}$ falls within the range of observed Arctic winter clear-sky downwelling longwave fluxes, and $F_{\text{cloudy}}^{\text{bb}}\approx 255Wm^{-2}$ corresponds to cloud-base blackbody emission near −15°C. Bootstrap resampling yields approximately 10% parameter uncertainty.

The fitted $\kappa_{\text{eff}}\approx 0.045{\left(gm^{-2}\right)}^{-1}$ is smaller than the monochromatic absorption coefficients reported for narrow spectral bands [0.058 at λ = 8 μm to 0.093 at λ = 11.4 μm (*49*)], as expected for the broadband, spectrally integrated coefficient defined above. The shape of the diffusion closure $D_{\text{LWP}}\left(x\right)$ depends only on $\kappa_{\text{eff}}$, whereas $F_{\text{max}}$ rescales the forcing amplitude $g^{2}$ and is absorbed into the prefactor $k_{\text{LW}}$.

#### LWP-based diffusion closure

Using the linearized mapping (Eq. 8) and the noise-amplitude function g(x) (Eq. 9), we represent the LWP contribution to the diffusion as $D_{\text{LWP}}\left(x\right)=k_{\text{LW}}g^{2}\left(x\right)$ (Eq. 11). The prefactor $k_{\text{LW}}$ (hour^−1^) is calibrated empirically and absorbs unresolved correlation-time effects in δLWP together with uncertainty in the broadband transfer-function parameters. The state dependence of the closure is therefore carried by $g^{2}\left(x\right)={\left[S_{\text{LW}}\sigma_{\text{LWP}}\right]}^{2}$. We next describe the estimation procedure.

Empirically, $\sigma_{\text{LWP}}\approx 8.4\sqrt{\text{LWP}}$ (slope 95% CI [7.6 to 8.5]; $R^{2}=0.97$ [0.89 to 0.98]; Fig. 4A), consistent with a Poisson-like scaling where variability grows with the square root of the mean cloud thickness. Because the bin-by-bin variance estimate is inherently noisier than the mean, we smooth $\sigma_{\text{LWP}}\left(x\right)$ with a Gaussian kernel (SD = 7 bins) before evaluating the closure. Conversely, we evaluate $S_{\text{LW}}$ directly from the unsmoothed conditional mean LWP without additional smoothing. Without smoothing $\sigma_{\text{LWP}}$, the shape correlation between $D_{\text{LWP}}$ and $D_{\text{obs}}$ drops from r=0.96 [0.60 to 0.96] to r=0.78 [0.13 to 0.81].

#### Seasonal diffusion decomposition

We fit the two-term closure (Eq. 15) via non-negative least squares (NNLS) over the sampled *x* range, enforcing $k_{\text{LW}},k_{\text{SW}}\geq 0$. In DJF (minimal sunlight), NNLS recovers $k_{\text{LW}}\approx 0.0178{\mathrm{hour}}^{-1}$ and $k_{\text{SW}}\approx 0$, consistent with Eq. 11.

The longwave forcing amplitude $g_{\text{LW}}\left(x\right)\equiv S_{\text{LW}}\left(x\right)\sigma_{\text{LWP}}\left(x\right)$ is computed using the DJF-diagnosed transfer-function parameters ($\kappa_{\text{eff}}$, $F_{\text{max}}$). We only recompute the conditional LWP statistics [$\bar{\text{LWP}}\left(x\right)$, $\sigma_{\text{LWP}}\left(x\right)$] for each season. This approach assumes the fitted transfer-function parameters provide a leading-order broadband mapping from LWP to surface $F_{\text{LW},\text{down}}$ that varies weakly across seasons. For the shortwave forcing amplitude, we define cloud transmissivity $T\equiv F_{\text{SW},\text{down}}/F_{\text{SW},\text{clear}}$, estimating $F_{\text{SW},\text{clear}}\left(t\right)$ as the 98th percentile of $F_{\text{SW},\text{down}}$ within each (day-of-year, hour) group. The shortwave noise amplitude is then $\sigma_{\text{SW}}\left(x\right)\equiv F_{\text{SW},\text{clear}}\sigma_{T}\left(x\right)$, where $\sigma_{T}\left(x\right)\equiv \text{std}\left[T|x\right]$ and $F_{\text{SW},\text{clear}}$ is the bin-mean clear-sky shortwave flux.

The jointly fitted $k_{\text{LW}}$ varies only moderately across seasons (0.0178 hour^−1^ in DJF, 0.0126 hour^−1^ in MAM, 0.0129 hour^−1^ in JJA, and 0.0134 hour^−1^ in SON), remaining within ∼30% of its DJF value; the residual variation partly reflects redistribution between the two terms in the NNLS fit when both channels are active, and it may also reflect changes in the LWP decorrelation timescale across seasons. We interpret the SON result with caution, given its lower overall fit quality ($R^{2}=0.59$). For the seasonal split in Fig. 5D, we divide the explained variance between the two pathways in proportion to their mean fitted magnitudes, $k_{\text{LW}}\langle g_{\text{LW}}^{2}\rangle$ and $k_{\text{SW}}\langle \sigma_{SW}^{2}\rangle$. Because the two amplitudes are correlated, this split shares overlapping variance between the pathways and does not measure their independent contributions.

Formal model selection supports the two-term closure in sunlit seasons (March to November). The Bayesian information criterion (BIC) decisively favors the two-term model over either single-term alternative (ΔBIC<−35 in all cases). In DJF, the BIC prefers the one-term $D_{\text{LWP}}$ model (ΔBIC=+7 for adding $k_{\text{SW}}$), and bootstrap resampling (*N* = 1000) drives $k_{\text{SW}}$ to zero in 100% of replicates. Bootstrap 95% confidence intervals on the fitted prefactors are narrow relative to the point estimates (e.g., JJA: $k_{\text{LW}}=0.013$[0.012 to 0.013], $k_{\text{SW}}=0.023$[0.022 to 0.024] hour^−1^), indicating stable parameter identification when the relevant forcing pathway is active.

### Barrier-based regime diagnostics

#### Conditional empirical potential landscapes

To assess how background meteorological conditions organize the radiative regime structure, we construct conditional empirical potentials for the state variable $x\equiv F_{\text{LW},\text{net}}$ as a function of a conditioning variable *z*. Here, *z* denotes either the 2-m air temperature $T_{2m}$ or the surface pressure $P_{\text{sfc}}$, using the wintertime (DJF) Utqiaġvik observational record spanning 1998–2025, resampled to 10 min.

For each conditioning variable, we divide the wintertime record into quantile-based bins (18 bins for $T_{2m}$ and 14 bins for $P_{\text{sfc}}$), requiring at least 200 samples per bin. Within each bin $z_{i}$, we estimate the conditional PDF $p\left(x|z_{i}\right)$ using Gaussian kernel density estimation with bandwidth $h_{\text{KDE}}=0.1$ in units of the standardized variable. The corresponding conditional empirical potential is defined as

$$U\left(x|z_{i}\right)\equiv -\text{ln}p\left(x|z_{i}\right)$$ (26)

and is shifted such that $U_{\text{min}}=0$ within each bin.

Potential wells are identified from local maxima of $p\left(x|z_{i}\right)$ that exceed 25% of the bin-wise PDF maximum. These maxima are classified into clear and cloudy branches using the threshold $x=-28Wm^{-2}$ from the SDE estimated barrier (Fig. 2D), with clear states defined by $x<-28Wm^{-2}$ and cloudy states by $x\geq -28Wm^{-2}$. When two branches coexist, the barrier height is computed as the maximum of $U\left(x|z_{i}\right)$ between the corresponding well locations.

#### Regime classification and transition detection

The variance-decomposition requires assigning each hourly observation to a radiative regime and identifying discrete regime-transition events. We classify each hourly observation as clear ($F_{\text{LW},\text{net}}<-28Wm^{-2}$) or cloudy ($F_{\text{LW},\text{net}}\geq -28Wm^{-2}$) using the same effective-potential barrier defined above (Fig. 2D). A barrier-crossing event is identified when two consecutive hourly observations fall in opposite regimes: a clear hour followed by a cloudy hour (clear-to-cloudy), or the reverse (cloudy-to-clear). To ensure temporal continuity, crossings are counted only when the interval between successive valid observations does not exceed 2 hours; apparent transitions across longer data gaps are discarded.

Over the 22 DJF seasons with MWR LWP coverage (“Observational, reanalysis, and model datasets” section), this procedure yields 1550 clear-to-cloudy and 1601 cloudy-to-clear crossings (4650 and 4803 pretransition hours in the 1- to 3-hour window preceding each crossing). This pretransition window captures the period in which any systematic meteorological precursor to regime transition would be detectable. Barrier crossings include both sustained regime transitions and brief excursions (median duration 4 hours).

#### Hierarchical variance decomposition

To quantify how much of the hourly radiative variability preceding regime transitions can be explained by concurrent meteorological and cloud-related covariability, we perform a hierarchical variance decomposition targeting the hourly increment

$$\Delta F_{\text{LW},\text{net}}\left(t\right)\equiv F_{\text{LW},\text{net}}\left(t\right)-F_{\text{LW},\text{net}}\left(t-1\text{hour}\right)$$ (27)

Because $F_{\text{LW},\text{net}}$ responds diagnostically, rather than prognostically, to the instantaneous atmospheric state, concurrent meteorological and microphysical variations are the physically appropriate predictors. The analysis is performed separately for clear-to-cloudy and cloudy-to-clear pretransition hours, as defined in the “Regime classification and transition detection” section.

Predictor variables are organized into a five-level additive hierarchy:

1) Surface meteorology: temperature, pressure, wind speed, wind direction (represented by sin θ and cos θ), and relative humidity.

2) Lower-tropospheric meteorology: 700 hPa temperature, specific humidity, and vertical velocity ($\omega_{700}$) from ERA5, together with precipitable water vapor from ground-based MWR observations.

3) Surface turbulent fluxes: sensible and latent heat fluxes, approximated from surface observations using neutral-stability bulk aerodynamic formulae with infrared surface skin temperature from the colocated U.S. Climate Reference Network (USCRN) station (*50*).

4) LWP from MWR best-estimate retrieval.

5) Cloud-base properties: cloud-base height and cloud-base temperature from MWR retrieval.

At each hierarchy level, we include both the instantaneous value and the concurrent hourly tendency, Δx≡x(t)−x(t−1hour), of every predictor, computed on the full hourly time series before regime filtering. This yields up to 30 features at the final level.

For each hierarchy level, we train an XGBoost gradient-boosted tree regressor (*27*) with 50 trees, maximum depth 4, learning rate 0.1, and minimum child weight 20. Model skill is evaluated using leave-one-season-out cross-validation across the 22 DJF seasons with MWR coverage. The reported $R^{2}$ is the mean across held-out seasons, and 95% confidence intervals are estimated by bootstrap resampling the per-season $R^{2}$ values (5000 replicates). As a sensitivity test, we repeat the full hierarchy using ordinary linear regression and compare it with the level-only feature set (fig. S7).

We further apply the same hierarchical framework to MOSAiC central Arctic observations (DJF 2019–2020), using fivefold temporal cross-validation and an expanded predictor set that includes directly measured colocated surface fluxes, ice water path, and cloud condensation nuclei concentrations, where available (fig. S7, B and D).
