# Supplementary material for: Stochastic cloud fluctuations drive Arctic winter radiative bistability
Source: Sci Adv. 2026 Sep 30;12(40):eaeh3823. doi: 10.1126/sciadv.aeh3823 (PMC13626087; doi:10.1126/sciadv.aeh3823)
Supplement: Supplementary file 1 [file sciadv.aeh3823_sm.pdf]

Science Advances
AAAS

# Supplementary Materials for

## Stochastic cloud fluctuations drive Arctic winter radiative bistability

Jung-Sub Lim and Graham Feingold

Corresponding author: Jung-Sub Lim, jung-sub.lim@noaa.gov

*Sci. Adv.* **12**, eaeh3823 (2026)
DOI: 10.1126/sciadv.aeh3823

**This PDF file includes:**

Figs. S1 to S12

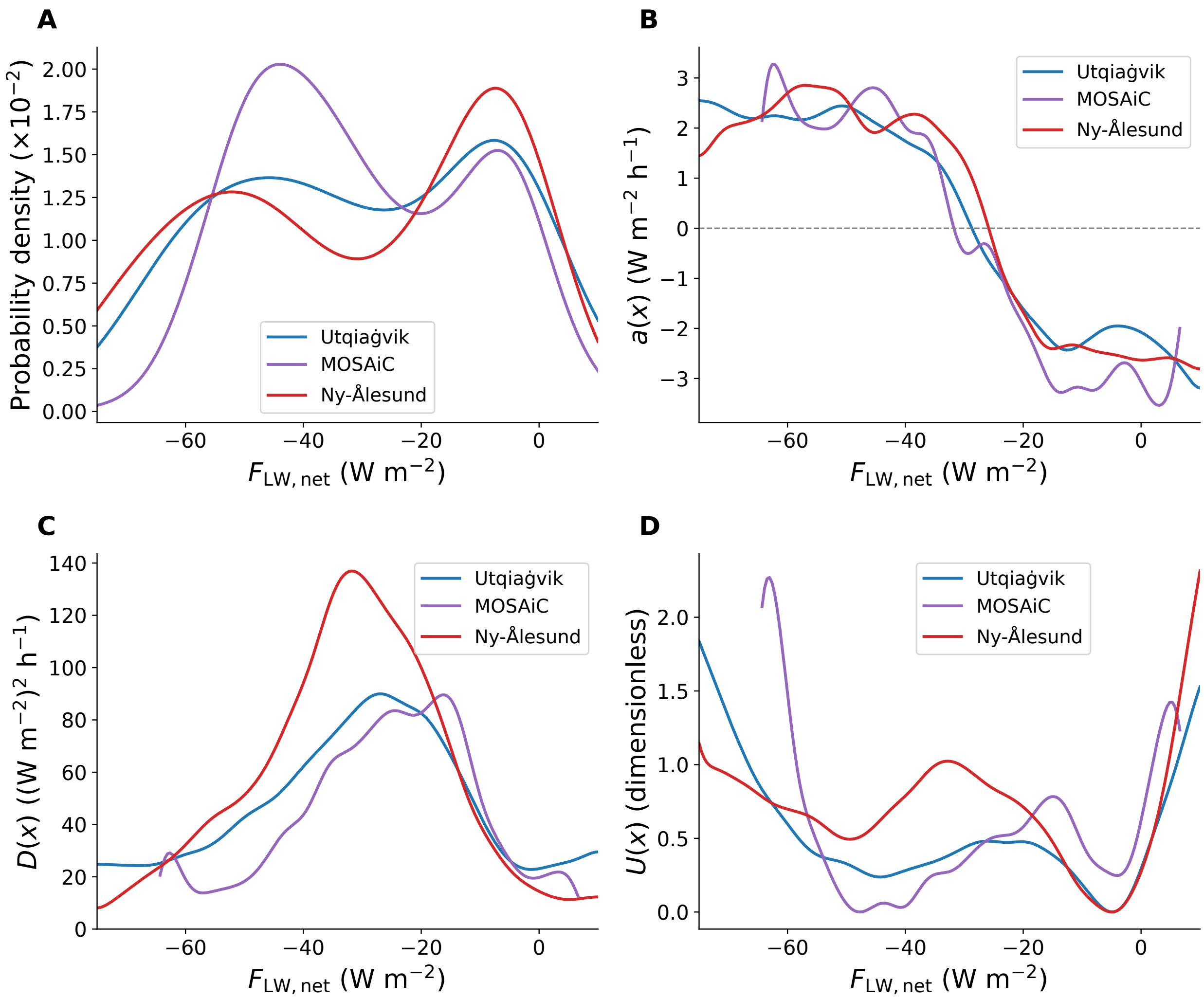


**Figure S1**: **Multi-location SDE comparison.** **(A)** PDF, **(B)** effective drift $a(x)$, **(C)** diffusion $D(x)$, and **(D)** effective potential $U(x)$ estimated independently from observations at Utqiaġvik, MOSAiC (central Arctic), and Ny-Ålesund (Svalbard). All three sites exhibit qualitatively similar drift–diffusion structure and corresponding double-well effective potentials, supporting the generality of the bistability mechanism.

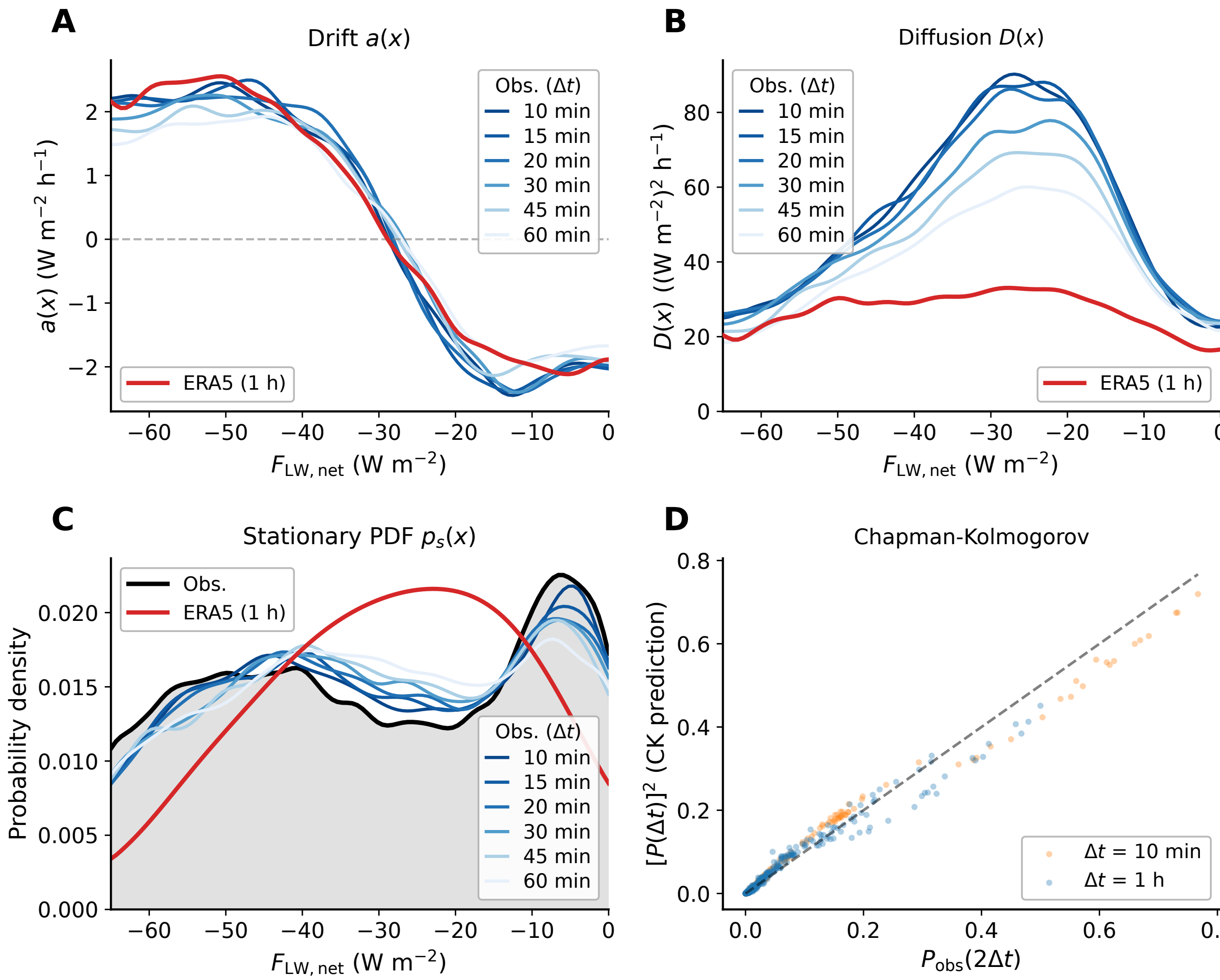


**Figure S2**: **SDE estimation validation and matched-resolution comparison with ERA5. (A)** Effective drift $a(x)$ and **(B)** diffusion $D(x)$ for the observations estimated at six temporal lags ($\Delta t$ = 10–60 min, blue); the drift curves closely overlap across all lags, and the diffusion magnitude decreases at coarser resolution, as expected from temporal averaging, while its peaked shape is retained. For a matched-resolution comparison, ERA5 at its native 1-h resolution is overlaid (red): its drift agrees with the observations, but its diffusion lacks the transition-range peak. **(C)** Stationary PDF $p_{\mathrm{st}}(x)$ reconstructed from the estimated $a(x)$ and $D(x)$ at each $\Delta t$ (blue) compared with the empirical PDF of the observations (black); the observed PDF is bimodal across all lag choices, whereas the ERA5 reconstruction (red) is unimodal. **(D)** Chapman–Kolmogorov consistency: scatter of observed $2\Delta t$ transition probabilities versus the Markov prediction $[P(\Delta t)]^2$ at $\Delta t$ = 10 min (orange) and $\Delta t$ = 1 h (blue), supporting the Markov approximation.

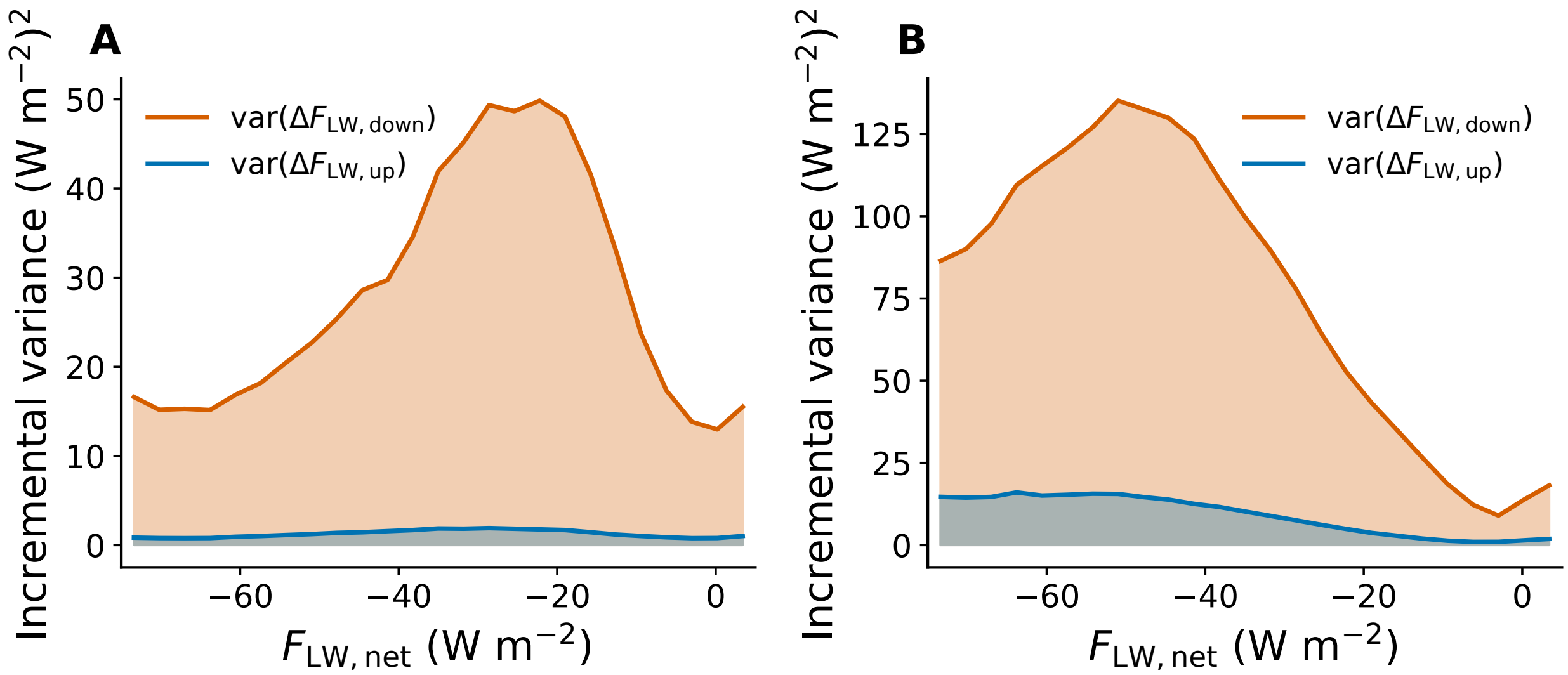


**Figure S3**: **Sub-hourly variance decomposition of $F_{\mathrm{LW,net}}$.** State-dependent incremental variance at $\tau$ = 10 min for $F_{\mathrm{LW,down}}$ (orange) and $F_{\mathrm{LW,up}}$ (blue), binned by $F_{\mathrm{LW,net}}$. **(A)** DJF. **(B)** JJA. In both seasons, sub-hourly variance is dominated by $F_{\mathrm{LW,down}}$.

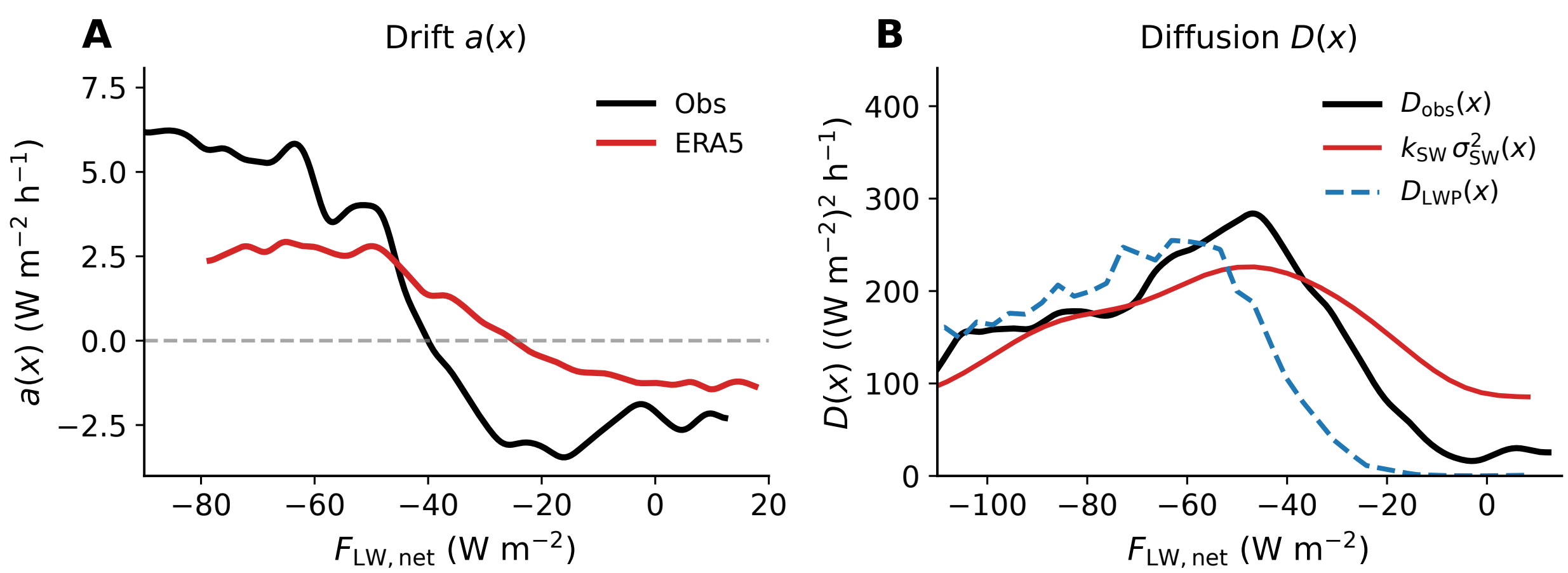


**Figure S4**: **Summer (JJA) drift and diffusion. (A)** Effective drift $a(x)$ estimated from June–August observations (black) and ERA5 (red). **(B)** Observed summer diffusion $D_{\mathrm{obs}}(x)$ (black) compared with the scaled shortwave contribution $k_{\mathrm{SW}}\,\sigma^2_{\mathrm{SW}}(x)$ (red; $R^2$ = 0.69) and the LWP-based closure $D_{\mathrm{LWP}}(x)$ (blue dashed; $R^2$ = 0.33).

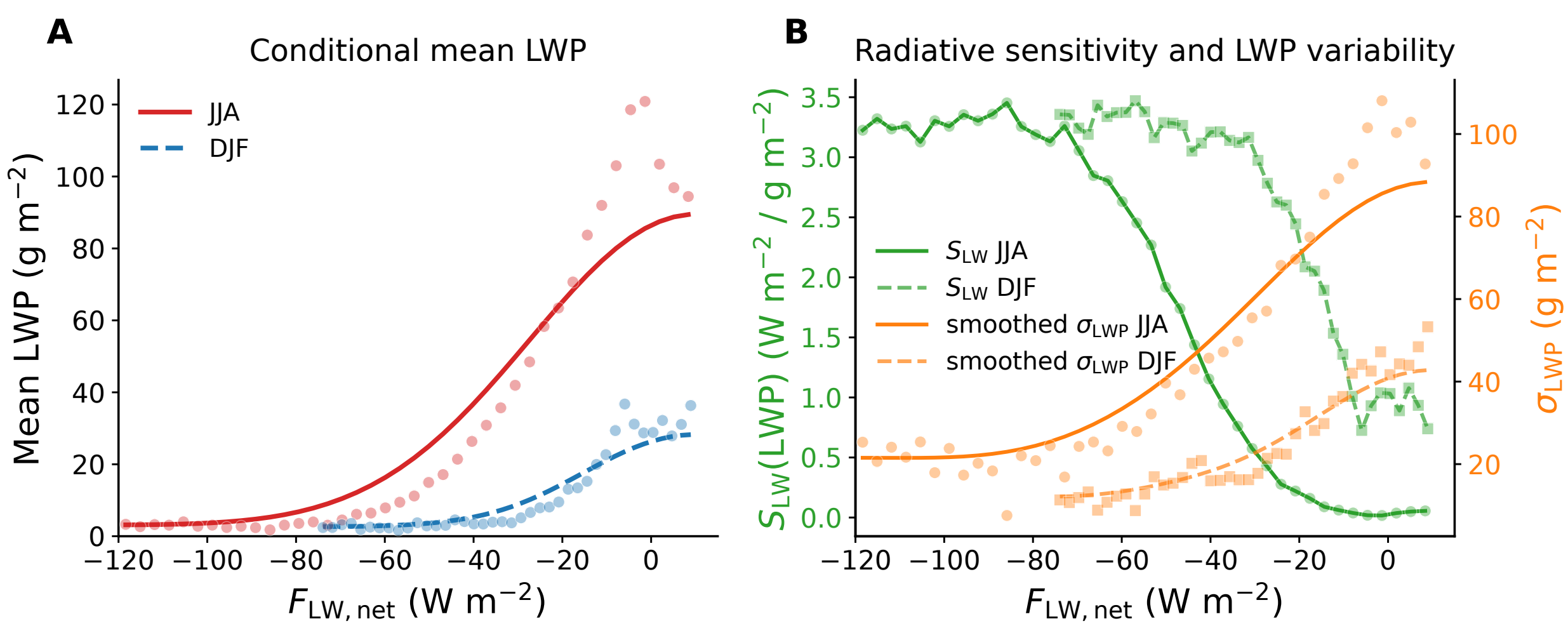


**Figure S5**: **Seasonal contrast in the LWP-based diffusion closure.** **(A)** Conditional mean LWP as a function of $F_{\mathrm{LW,net}}$ for JJA (red) and DJF (blue dashed), showing that summer clouds are systematically thicker. **(B)** Radiative sensitivity $S_{\mathrm{LW}}$ (green; left axis) and LWP variability $\sigma_{\mathrm{LWP}}$ (orange; right axis) for both seasons.

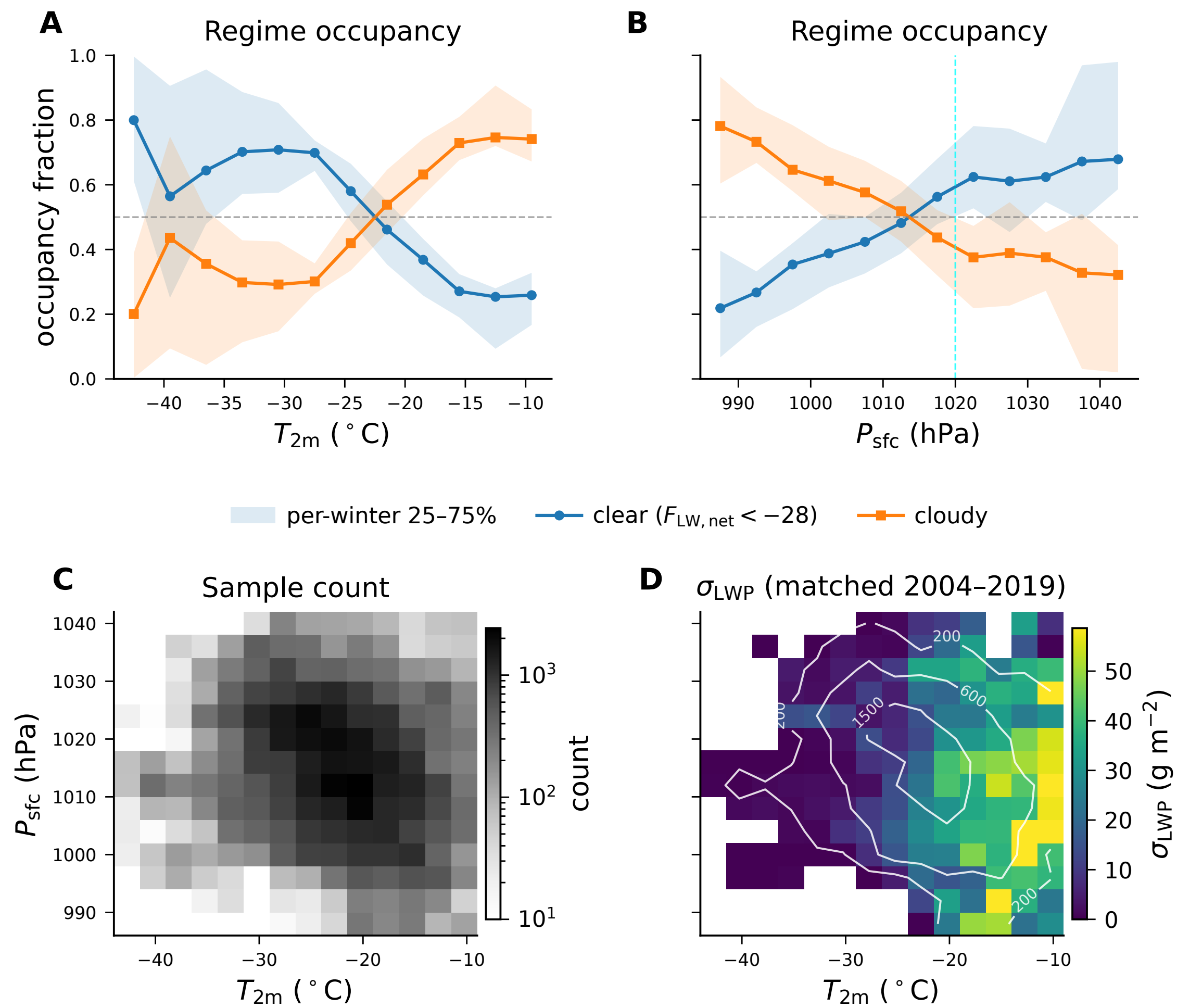


**Figure S6**: **Regime occupancy, sampling, and cloud-liquid variability under meteorological conditioning. (A), (B)** Clear- and cloudy-regime occupancy fractions (clear: $F_{\mathrm{LW,net}} < -28$ W m$^{-2}$) versus $T_{2\mathrm{m}}$ **(A)** and $P_{\mathrm{sfc}}$ **(B)**, with per-winter interquartile spread (shading). **(C)** 10-min sample count on the $(T_{2\mathrm{m}}, P_{\mathrm{sfc}})$ plane. **(D)** Cloud-liquid variability $\sigma_{\mathrm{LWP}}$ on the same plane (matched $F_{\mathrm{LW,net}}$–LWP record, DJF 2004–2019).

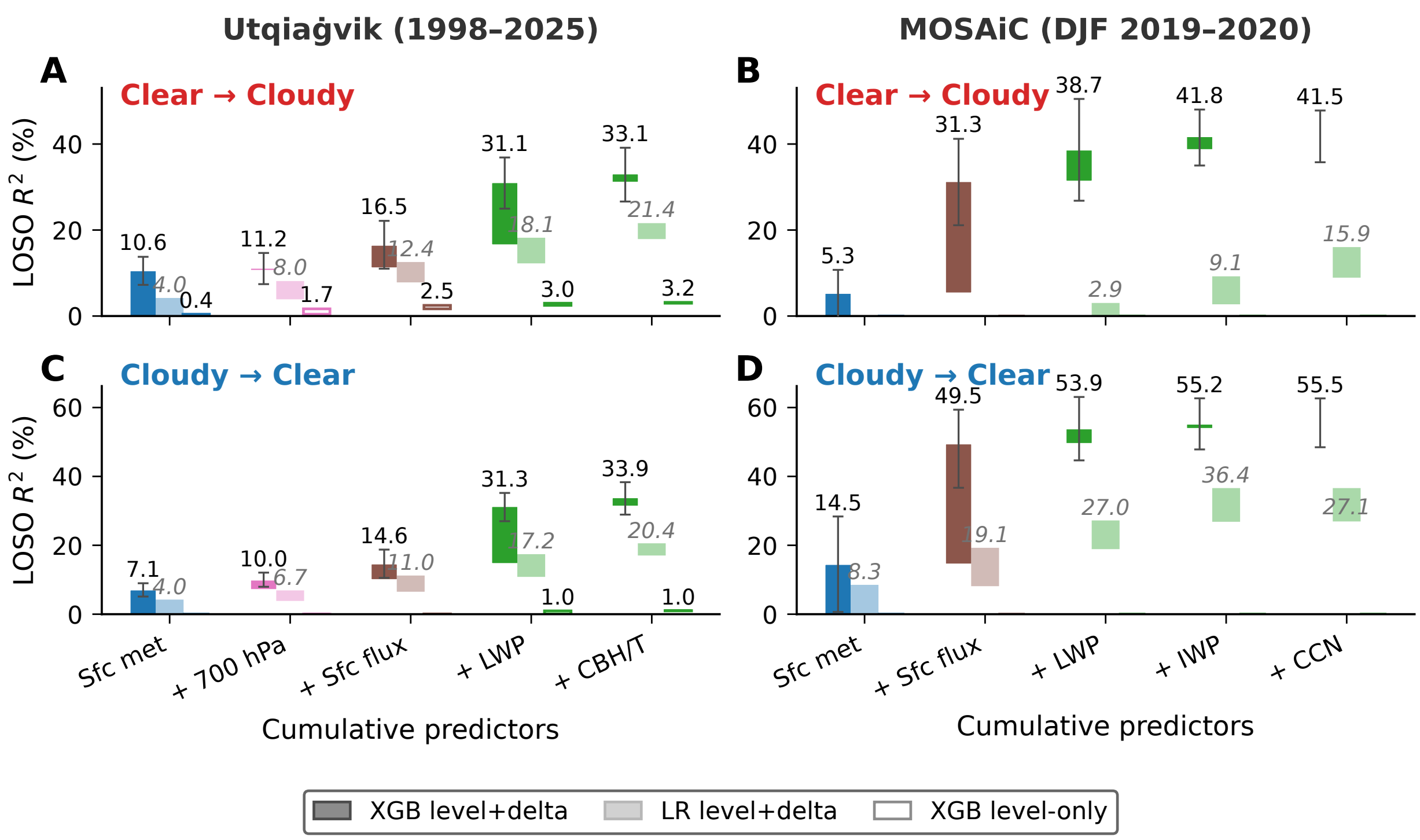


**Figure S7**: **Sensitivity of variance decomposition to model choice and site.** Hierarchical decomposition comparing XGBoost with level+Δ features (solid bars), ordinary linear regression with level+Δ features (lighter bars), and XGBoost with level-only features (open bars). Here, "level" denotes the instantaneous value of each predictor variable, whereas Δ denotes its hourly tendency, computed as the one-hour change in that variable. Panels **A,C** show results from Utqiaġvik (22 MWR-valid DJF seasons), whereas panels **B,D** show results from MOSAiC (DJF 2019–2020; 5-fold temporal cross-validation). Panels **A,B** correspond to clear-to-cloudy transitions, and panels **C,D** to cloudy-to-clear transitions.

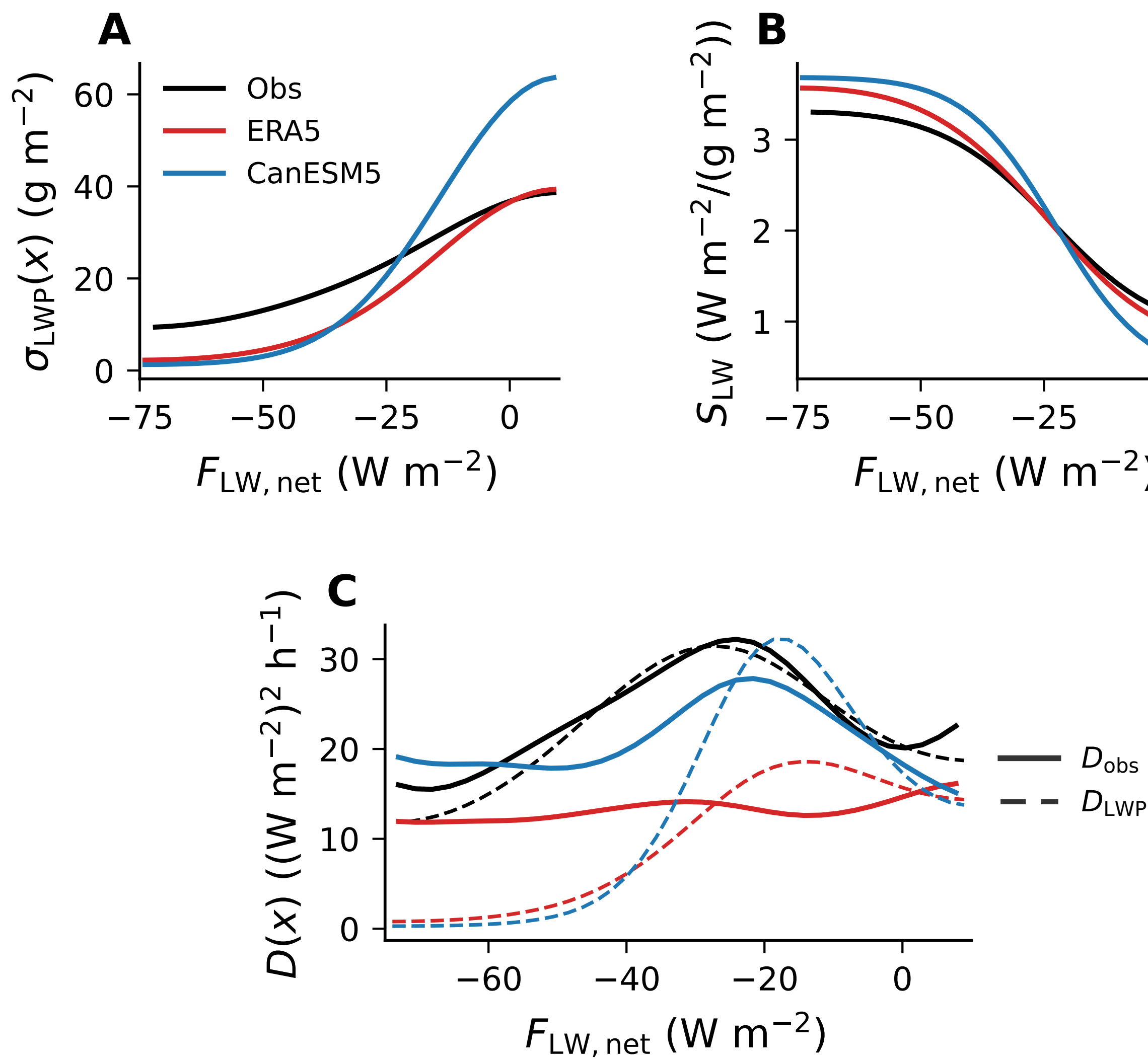


**Figure S8**: **LWP-based diffusion closure for observations, ERA5, and CanESM5 AMIP at matched 3-hourly resolution.** **(A)** Cloud-liquid variability $\sigma_{\rm LWP}(x)$. **(B)** Radiative sensitivity $S_{\rm LW}(x)$ from the Beer–Lambert form of Eq. (25). **(C)** Kramers–Moyal diffusion $D(x)$ (solid) and the LWP-based closure prediction $D_{\rm LWP}(x)$ (dashed). Line colors indicate observations (black), ERA5 (red), CanESM5 (blue).

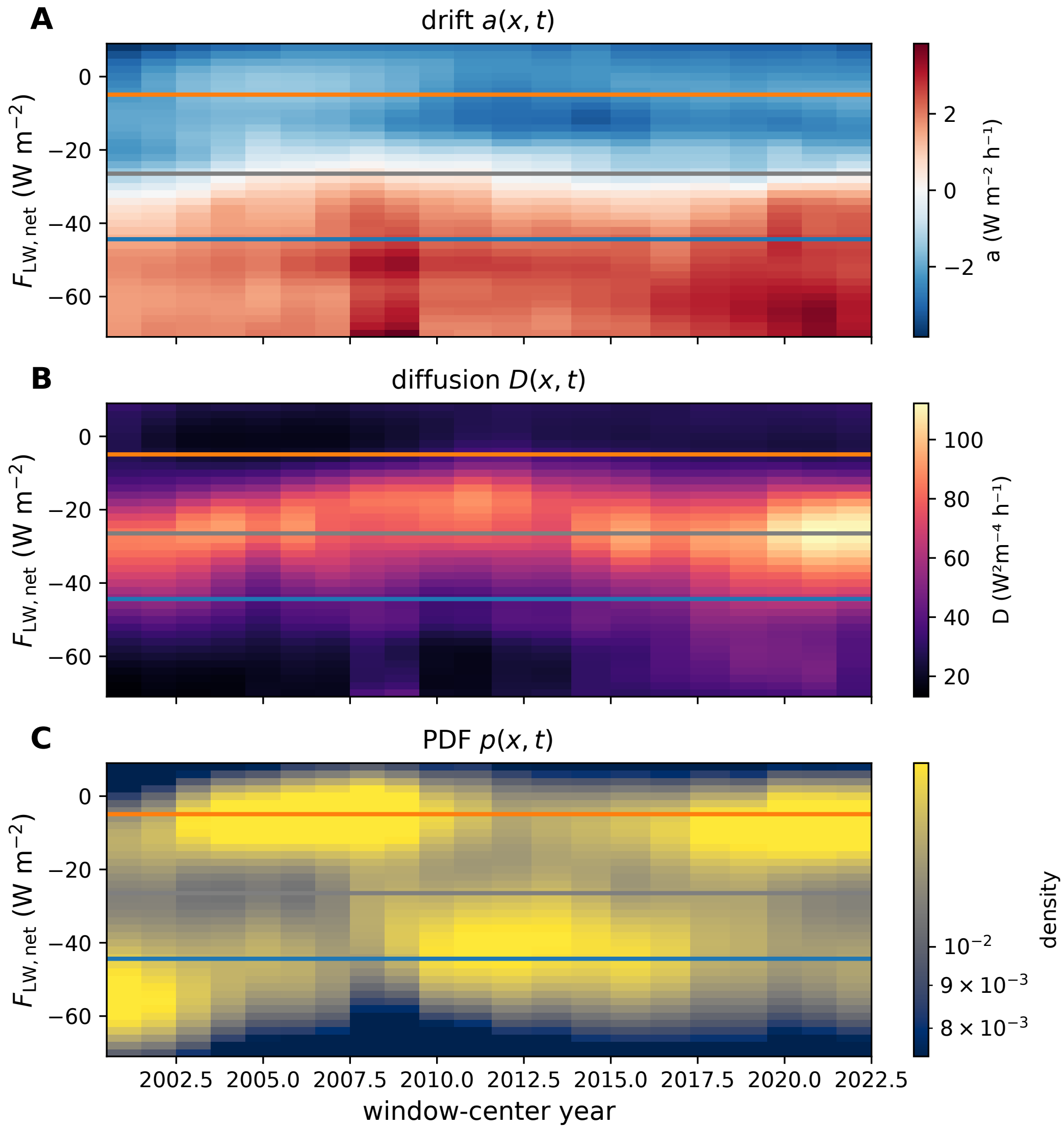


**Figure S9**: **Moving-window Kramers–Moyal diagnostic of the reduced radiative dynamics.** The drift $a(x)$ (**A**) and diffusion $D(x)$ (**B**) re-estimated over sliding 7-winter windows across the Utqiaġvik DJF record (1998–2025), and the window-wise empirical PDF (**C**). Horizontal lines mark the clear well, the barrier, and the cloudy well.

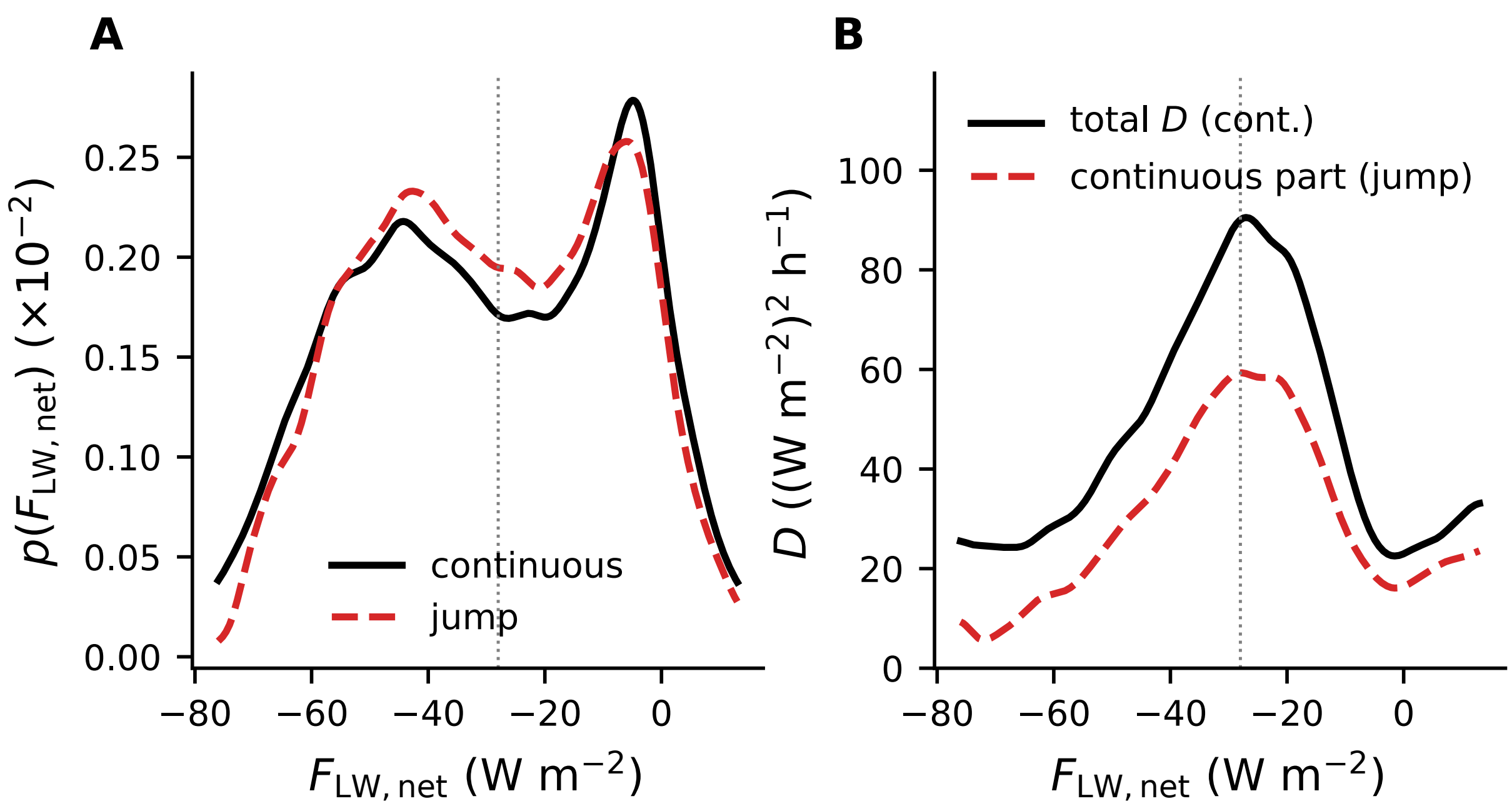


**Figure S10**: **Continuous versus finite-variance jump-diffusion decomposition.** **(A)** Reconstructed stationary density for the continuous Fokker–Planck model (solid) and an admissible finite-variance jump-diffusion (dashed); the two differ by 4.9% $[3.6, 6.7]$ in total variation. **(B)** Total Kramers–Moyal diffusion (solid) and the continuous part remaining after the jump contribution is removed (dashed): the transition-range peak is preserved with reduced magnitude. Data are derived from Utqiaġvik for DJF at 10-min resolution.

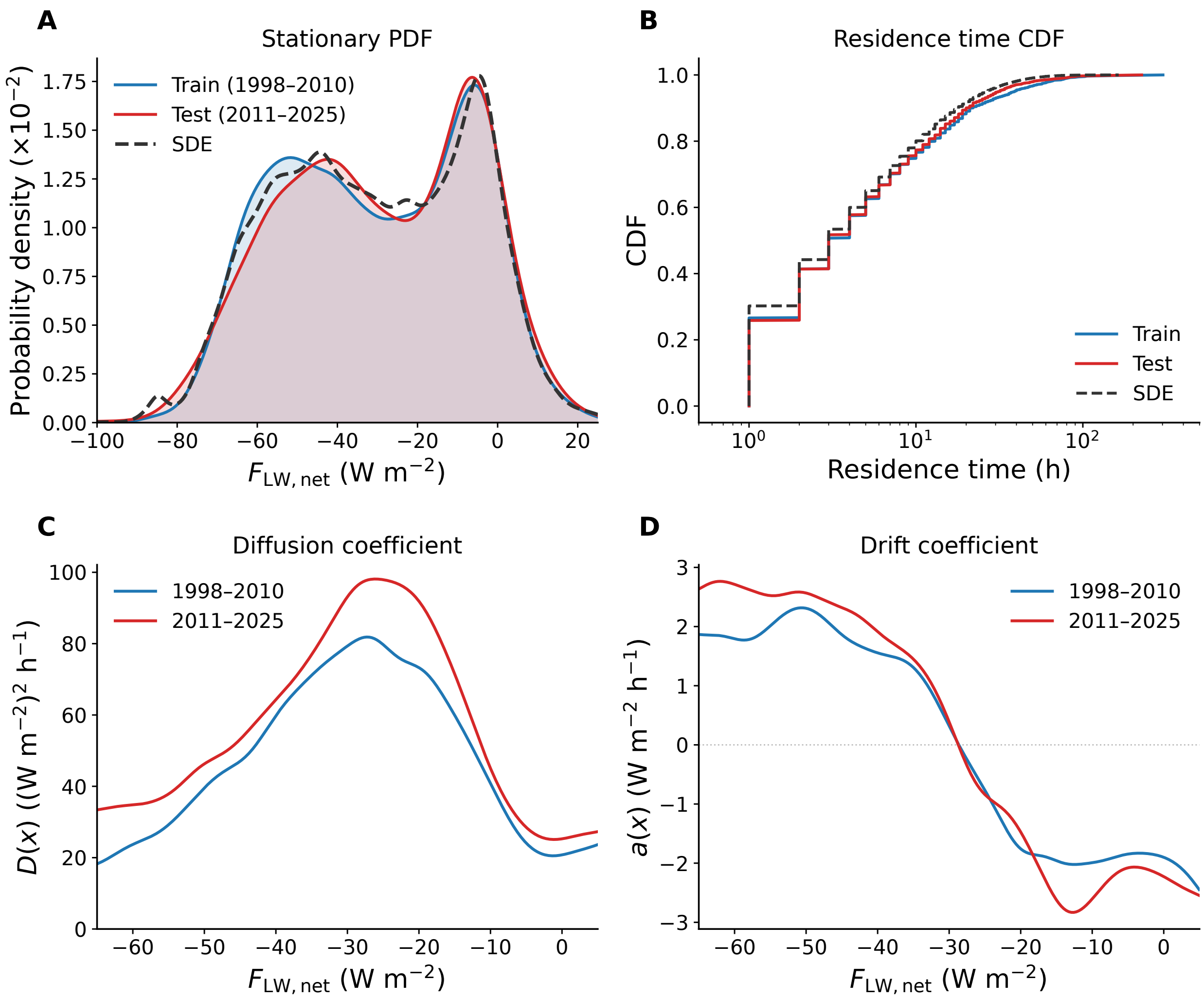


**Figure S11**: **Out-of-sample validation. (A)** Stationary PDF: the SDE trained on 1998–2010 reproduces the 2011–2025 test distribution ($r = 0.99$). **(B)** Residence-time CDFs for the training period, test period, and SDE-predicted series. **(C)** Diffusion $D(x)$ and **(D)** effective drift $a(x)$ estimated independently from the training and test periods, showing high temporal stability ($r > 0.98$ for both coefficients).

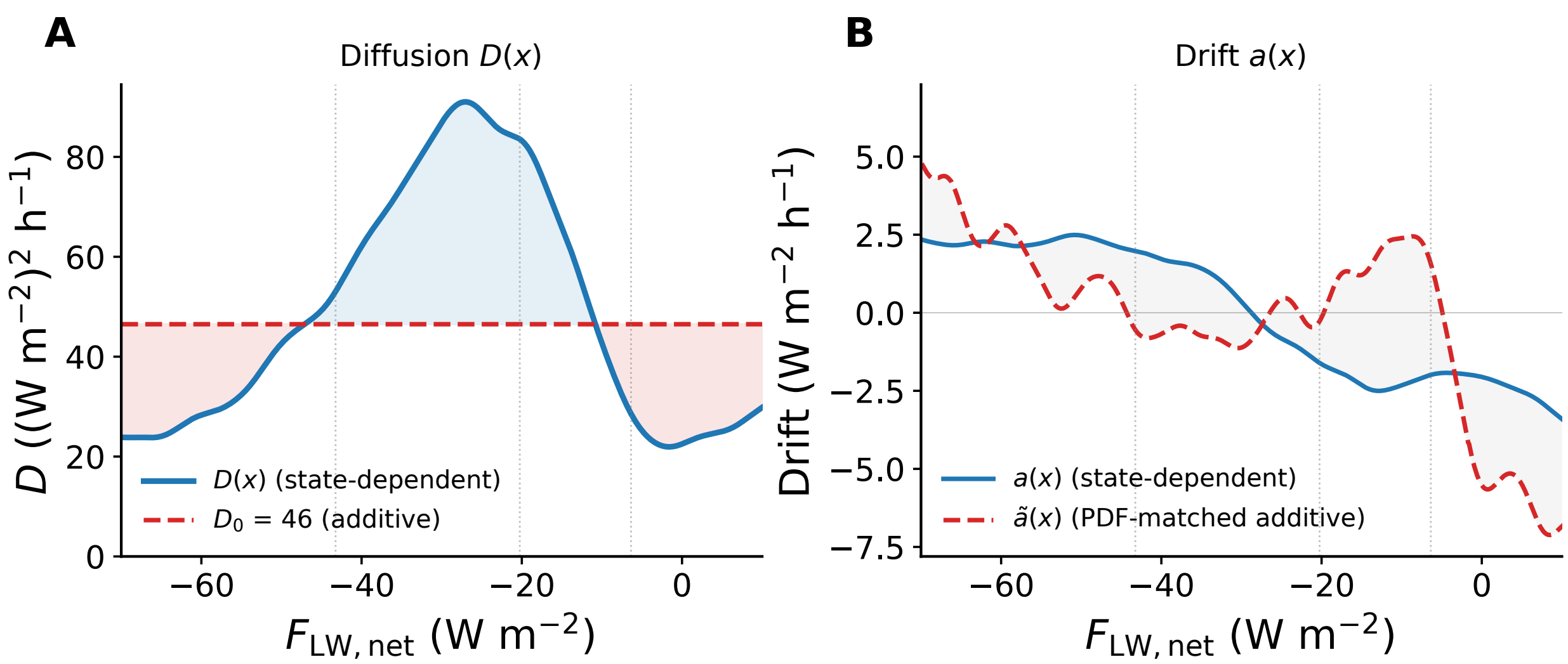


**Figure S12**: **State-dependent vs. additive noise. (A)** Diffusion coefficient $D(x)$ (blue) compared with the constant $D_0 = \langle D \rangle$ assumed by the additive model (red dashed). **(B)** Drift functions for the state-dependent model $a(x)$ (blue) and the PDF-matched additive model $\tilde{a}(x)$ (red dashed); both yield identical stationary PDFs by construction.
